# Experimental and theoretical evaluation on the antioxidant activity of a copper(ii) complex based on lidocaine and ibuprofen amide-phenanthroline agents†

**DOI:** 10.1039/c8ra09763a

**Published:** 2019-01-25

**Authors:** Leila Tabrizi, Duy Quang Dao, Thuy An Vu

**Affiliations:** School of Chemistry, National University of Ireland, Galway University Road Galway H91 TK33 Ireland leila.tabrizi@nuigalway.ie; Institute of Research and Development, Duy Tan University 03 Quang Trung Da Nang 550000 Viet Nam daoduyquang@gmail.com; Faculty of Environment and Chemical Engineering, Duy Tan University 03 Quang Trung Da Nang 550000 Viet Nam

## Abstract

A new copper(ii) complex, [Cu(LC)(Ibu-phen)(H_2_O)_2_](ClO_4_)_2_ (LC: lidocaine, Ibu-phen: ibuprofen amide-phenanthroline), was synthesized and characterized. The antioxidant activities of the free ligands and the copper(ii) complex were evaluated by *in vitro* experiments and theoretical calculations using density functional theory (DFT). Structures of the ligand Ibu-phen and the complex were identified by ^1^H and ^13^C NMR, FT-IR spectroscopies, mass spectrometry, thermogravimetric analysis and elemental analysis. The antioxidant potentials of LC and Ibu-phen ligands as well as copper(ii) complex were also evaluated by DPPH˙, ABTS˙^+^, HO˙ essays and EPR spectroscopy. The experimental results show that the radical scavenging activity (RSA) at various concentrations is decreased in the following order: copper(ii) complex > ascorbic acid > LC > Ibu-phen. Structural and electronic properties of the studied compounds were also analyzed by DFT approach at the M05-2X/6-311++g(2df,2p)//M05-2X/LanL2DZ level of theory. ESP maps and NPA charge distributions show that the highly negative charge regions found on the N and O heteroatoms make these sites more favorable to bind with the central copper ion. Frontier orbital distributions of copper(ii) complex indicate that HOMOs are mainly localized at Ibu-phen, while its LUMOs are distributed at LC. Based on natural bond orbitals (NBO) analyses, Cu(ii) ion plays as electron acceptor in binding with the two ligands and two water molecules. Thermochemical properties including bond dissociation enthalpy (BDE), ionization energy (IE), electron affinity (EA), proton affinity (PA) characterizing three common antioxidant mechanisms *i.e.* hydrogen transfer (HT), single electron transfer (SET) and proton loss (PL) were finally calculated in the gas phase and water solvent for two ligands and the copper(ii) complex at the same level of theory. As a result, the higher EA and lower BDE and PA values obtained for copper(ii) complex show that the complex shows higher antioxidant potential than the free ligands.

## Abstract 2

The copper(ii) complex of lidocaine and ibuprofen amide-phenanthroline was synthesized and characterized in order to study antioxidant activity by experimental and theoretical evaluations.

## Graphical abstract

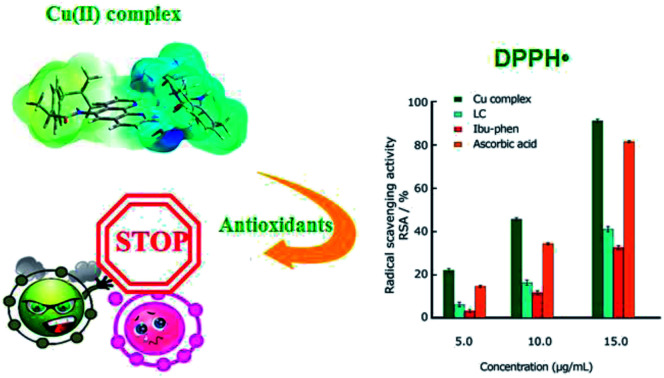

## 1 Introduction

Oxidative stress (OS) is a health-threatening process that is involved, at least partially, in the development of several human diseases including different types of atherosclerosis, inflammatory injuries, cardiovascular diseases, cancer, neurodegenerative diseases, and aging.^1–5^ It can be defined as an imbalance between reactive oxygen species (ROS) and antioxidant levels leading to cell damage and health problems. OS provokes the production of ROS which are generally oxygen-containing radical species such as superoxide anion radicals, hydroxyl radicals or even hydrogen peroxide and singlet oxygen. Reactive nitrogen species (RNS), reactive sulfur species (RSS) could be also generated during oxidative stress. Antioxidants moderate ROS levels in cells and can therefore attend as a type of defensive medicine for human diseases caused by OS.

The transition metal ions can endorse an extensive range of coordination numbers, geometries, and oxidation states in comparison with other main group elements. The great potential value of finding or creating new antioxidant classes has already encouraged researchers to study for the metal-derived antioxidants. However, the antioxidant capacity of metal complexes is still not evident. Indeed, several experimental data in the literature reveals that flavonoid complexes are more effective radical scavengers than free flavonoids.^6^ The enhanced activity was reported for the Fe(ii), Fe(iii), Cu(ii) and Zn(ii) complexes of rutin, epicatechin and dihydroquercetin.^7,8^

On the other hand, the role of flavonoids as pro-oxidant agents, especially in cancer cell lines, has been the subject of research for decades.^9–12^ The complexes of flavonoid compounds with metals like Cu(ii) or Fe(iii) have been shown to act as pro-oxidant in forming hydroxyl radical. This trend can be explained by the fact that metal coordination changes the redox potential of ligand and thus affects on its antioxidant capacity.^13^ For example, luteolin (5,7,3′,4′-tetrahydroxyflavone) shows better antioxidant activity than the luteolin–Fe(iii) complex in the DPPH˙ assay.^14^ Marković *et al.* evaluate the relevant interactions of morin and quercetin, as well as their respective iron(iii) complexes with DPPH˙, tempone, hydroxyl and superoxide radicals.^15^ The authors observe that both quercetin and morin present higher free radical scavenging activity than their corresponding complexes with Fe(iii) ion.

Thus, several questions rise from, first, the choice of metal and linker to build the complex which promotes the enhanced antioxidant activity and second, the type of mechanism which favors the improvement of the antioxidant capacity of the metal complex compared to that of their parent components. The lack of an in-deep understanding about the general behavior of metal complexes in this field prompts us to study the antioxidant activity of metal complexes by both experimental and theoretical ways.

The main objective of this work consists of synthesizing a new copper(ii) complex based on lidocaine and ibuprofen amide-phenanthroline ligands which hopefully possesses stronger radical scavenging activity in comparison with its ligands, and evaluating their antioxidant activities through experimental assays and computational chemistry approach. It is noteworthy that while seeking for the development of metallodrugs as potent antioxidant agents, Cu(ii) ion appears to us as natural ideal metal candidate. The Cu(ii) is a biologically essential ion that includes positive redox potential in biological electron transfer reactions. Copper complexes have revealed significant performance in antioxidant studies.^16–21^ In addition, lidocaine (LC) is a local anesthetic agent widely used in the clinic therapy which is reported to act as a concentration dependent antioxidant.^22^ Recently, the syntheses and biological properties on the Ni(ii), Co(ii), Ru(ii), Ir(iii), Pt(ii) and Pd(ii) complexes with lidocaine have been reported for traditional chemotherapy and photodynamic therapy.^23–27^ Ibuprofen is a propionic acid derivative and nonsteroidal anti-inflammatory drug with anti-inflammatory, analgesic and antipyretic effects. Ibuprofen inhibits the activity of cyclo-oxygenase I and II (COX-1 and COX-2), causing a reduced formation of precursors of prostaglandins and thromboxanes.^28,29^

Herein, we report the synthesis and characterization of a new copper(ii) complex based on lidocaine and ibuprofen amide-phenanthroline agents. The antioxidant activity of the free ligands and copper complex are experimentally evaluated by DPPH˙, ABTS˙^+^ and HO˙ radicals scavenging assays. A density functional theory (DFT) study based on calculations of thermochemical parameters such as bond dissociation enthalpy (BDE), ionization energy (IE), electron affinity (EA) and proton affinity (PA) is also performed in the gas phase and water solvent. These parameters allow providing more insight into its free radical scavenging capacity and mechanism.

## 2 Methods

### 2.1. Materials and methods

The ligand Ibu-phen was synthesized under nitrogen atmosphere by standard Schlenk techniques using as-received analytical or HPLC grade solvents and reagents from commercial suppliers.^30^ 2,2′-Azino-bis(3-ethylbenzthiazoline-6-sulphonic acid) (ABTS˙^+^) and 2,2-diphenyl-1-picrylhydrazyl (DPPH˙), ascorbic acid and lidocaine were purchased from Sigma-Aldrich.

Fourier transform infrared (FT-IR) spectra were recorded on a PerkinElmer Spectrum 400 (FT-IR/FT-NIR spectrometer) fitted in the 650–3600 cm^−1^ range. ^1^H, ^13^C nuclear magnetic resonance (NMR) spectra were recorded on a Bruker-400 MHz spectrometer at ambient temperature in DMSO-*d*_6_. Electrospray ionization (ESI) mass spectra were recorded on a Waters LCT Premier XE spectrometer in positive- or negative-ion mode. Elemental analyses were performed with an EA 3000 CHNS. Electron paramagnetic resonance (EPR) spectra (X-band, 0.34 T, 9.5 GHz) were obtained with a Varian spectrometer, equipped with a variable-temperature facility in the following conditions: 3385.0 Gs field, 20.0 mV power, 100.0 kHz modulation frequency, 1.0 GS amplitude and 300 seconds sweep time. The *g* values were determined using a DPPH standard. Electronic absorption spectra were obtained on a Shimadzu Lambda-1600 UV-Vis spectrophotometer. Solid state UV-Vis diffused reflectance spectra were recorded with a Shimadzu 2450 PC UV-Vis recording spectrophotometer. Thermogravimetric analysis (TGA) of the copper(ii) complex was obtained on a STA625 thermal analyzer from Rheometric Scientific by collecting 10 mg of the compound in nitrogen atmosphere. The heating rate was kept constant at 10 °C min^−1^.

### 2.2. Synthesis of ibuprofen amide-phenanthroline (Ibu-phen)

To a solution of ibuprofen (0.661 g, 3.2 mmol) in DMF (10 mL) was added *N*,*N*-diisopropylethylamine (DIEA) (1.251 g, 9.7 mmol). The mixture was cooled to 0 °C and treated with *N*-(3-dimethylaminopropyl)-*N′*-ethylcarbodiimide hydrochloride (EDC-HCl) (1.2 g, 6.5 mmol), 1-hydroxybenzotriazole hydrate (HOBt·*x*H_2_O) (0.879 g, 6.5 mmol) and 1,10-phenanthrolin-5-amine (0.957 g, 4.9 mmol). The reaction was stirred at room temperature for 15 h under nitrogen. After completion, the mixture was diluted with water (50 mL) and extracted with dichloromethane (70 mL). The organic layer was dried with sodium sulfate (Na_2_SO_4_) and concentrated in vacuum. The resulting material was purified by silica gel column chromatography (3% methanol/dichloromethane) to provide the product as orange solids (1.058 g, 86% yield, 3.2 mmol). Anal. calc. (%) for C_25_H_25_N_3_O (383.1998): C, 78.30; H, 6.57; N, 10.96; found (%): C, 78.22; H, 6.51; N, 10.90. TOF-MS: 384.2076 [M + H]^+^. FT-IR: *ν* 3219 (NH stretch), 1679 (C=O stretch) cm^−1^. ^1^H NMR (DMSO-*d*_6_): *δ* 9.02 (s, 1H), 8.68 (m, 2H, H–Ar), 8.01 (m, 1H, H–Ar), 7.69 (m, 1H, H–Ar), 7.17 (t, 2H, H–Ar, *J* = 5.0 Hz), 7.08 (t, 2H, H–Ar, *J* = 5.0 Hz), 6.87 (s, 1H, H–Ar), 3.50 (q, 1H, H-6, *J* = 5.0 Hz), 2.36 (d, 2H, H-3, *J* = 10.0 Hz), 1.87 (m, 1H, H-2), 1.42 (d, 3H, H-7, *J* = 10.0 Hz), 0.90 (d, 6H, H-1, *J* = 10.0 Hz). ^13^C NMR (DMSO-*d*_6_): *δ* 174.9 (C=O), 160.0 (Ar), 152.2 (Ar), 148.1 (Ar), 142.3 (Ar), 136.0 (Ar), 133.1 (Ar), 129.9 (Ar), 128.3 (Ar), 125.7 (Ar), 123.9 (Ar), 122.1 (Ar), 44.6, 29.1, 24.3, 17.1.

### 2.3. Synthesis of the complex [Cu(LC)(Ibu-phen)(H_2_O)_2_](ClO_4_)_2_

A solution of Cu(ClO_4_)_2_·6H_2_O (0.37 g, 1 mmol) in acetonitrile (5 mL) was added to a solution of lidocaine (LC) (0.234 g, 1 mmol) in acetonitrile (5 mL). The mixture was stirred for 10 min, then a solution of ibuprofen amide-phenanthroline (Ibu-phen) (0.383 g, 1 mmol) in dichloromethane (10 mL) was added dropwise at 50 °C and the mixture was left stirring for 24 h. The mixture was filtered and the filtrate was dried under vacuum giving desired complex as a greenish-orange solid (660 mg, 72% yield, 1 mmol). Anal. calc. (%) for C_39_H_51_Cl_2_CuN_5_O_12_ (916.3005): C, 51.12; H, 5.61; N, 7.64; found (%): C, 51.07; H, 5.59; N, 7.59. TOF-MS: 815.2722 [M − (ClO_4_)]^+^. FT-IR: *ν* 3356 (H_2_O), 3227 (NH stretch), 1679, 1648 (C=O stretch), 1097 (ClO_4_^−^), 994 (ClO_4_^−^) cm^−1^.

**Warning**: perchlorate salts may be explosive.

### 2.4. DPPH˙, ABTS˙^+^ and HO˙ radicals scavenging assay

#### 2.4.1. DPPH˙

The DPPH˙ assay was carried out using the reported method with some modifications.^31^ To a 0.1 mM solution of DPPH˙ in MeOH (2 mL), was added a 5–15 μM solution of the inquired antioxidant in methanol (20 μL) and the reaction mixture was shaken vigorously. The reduction of DPPH˙ absorbance was followed by monitoring at 517 nm every 5 min for about 35 min. As a control, the absorbance of the blank solution of DPPH˙ (2 mL) was also registered at 517 nm.

#### 2.4.2. ABTS˙^+^

ABTS˙^+^ radical scavenging activity was based on the reported method.^32^ Briefly, ABTS powder (54.2 mg) was dissolved in 10 mL of phosphate buffer (5 mM, pH 7.0). 1.0 g of MnO_2_ was added and the resulting solution was incubated at room temperature for 30 min to generate a green color ABTS˙^+^ solution. The solution was centrifuged for 5 min and filtered to remove all excess of MnO_2_. The filtrate was diluted with phosphate buffer until the absorbance of solution measured at 723 nm equal to 0.70 ± 0.01 (final concentration 1 mM). Different concentrations (5–15 μM) of the inquired antioxidant (20 μL) were added to 2 mL of ABTS˙^+^ solution and incubated for 10 min at room temperature. The decrease of absorbance was monitored at 734 nm after 10 min.

#### 2.4.3. HO˙

Hydroxyl radical (HO˙) scavenging activity was investigated based on the method reported in literature.^33^ Briefly, different concentrations of (5–15 μM) of the inquired antioxidant (20 μL) were added to the reaction mixture, which contained 8 mM FeSO_4_ (0.25 mL), 6 mM H_2_O_2_ (0.4 mL), 0.25 mL distilled water and 20 mM sodium salicylate (0.1 mL) (final concentration 1 mM). Then the reaction system was incubated at 37 °C for 1 h. Absorbance value was determined at 562 nm.

For all assays, the radical scavenging activity (RSA) was calculated using the following equation:1
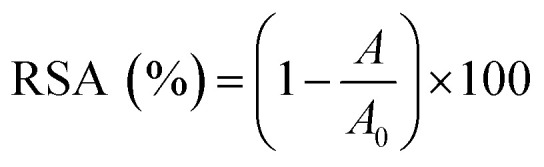
where *A*_0_ is the absorbance of the control, and *A* is the absorbance of test sample.

### 2.5. Electron paramagnetic resonance (EPR) measurements for DPPH˙, ABTS˙^+^ and HO˙ radicals scavenging assay

EPR spin trapping were applied to detect the DPPH˙ radical scavenging activity by LC, Ibu-phen, copper(ii) complex and ascorbic acid as previously described procedure.^34^ A solution of 5 μM or 15 μM of inquired antioxidant in EtOH (60 μL) was added to a 60 μM solution of DPPH in EtOH (60 μL). After mixing vigorously for 10 s, the solution was transferred into a 100 μL quartz capillary tube, and the spin adduct was measured after 2 min.

For ABTS˙^+^ radicals scavenging assay, a solution of 5 μM or 15 μM of inquired antioxidant in EtOH (50 μL) was added to a 50 μM solution of ABTS˙^+^ in EtOH (50 μL). After mixing vigorously for 5 s, the solution was transferred into a 100 μL quartz capillary tube, and the spin adduct was measured after 3 minutes.

For HO˙ scavenging assay, the Fenton reagents were used to test the ability of antioxidants.^35^ The reaction mixture contained 10 mM DMPO, 100 μM FeSO_4_, 10 mM H_2_O_2_ without and with the presence of each studied antioxidant. After stirring for 5 s, 50 μL of the mixture were transferred into a 100 μL disposal capillary tube. The EPR spectrum was recorded after 2.5 minutes.

The radical scavenging activity (RSA) was calculated using the following equation:2
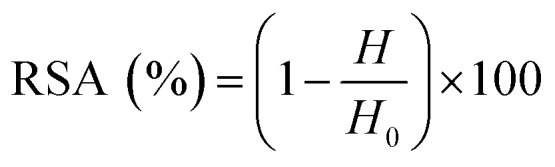
where *H* and *H*_0_ were the height of the third resonance peak for test sample and the control, respectively.

### 2.6. Statistical analyses

Obtained data are presented as averaged value ± standard deviation (SD). Statistical analyses were carried out using ANOVA and a Student's *t*-test and the Kruskal–Wallis and Mann–Whitney U-test (SPSS for Windows version 10.0). Differences were considered significant if *p* < 0.05.

## 3 Computational methods

All computational calculations were performed by employing GAUSSIAN 09 RevE.01.^36^ Geometry optimization and frequency calculations for LC, Ibu-phen and copper complex were investigated using M05-2X functional which has been suggested by its authors for estimation of thermodynamic parameters.^37^ All geometry optimizations were realized using the LanL2DZ basis set^38^ without any symmetry constraints.^39^ LanL2DZ is known as an appropriate basis set to describe electronic structure for compounds of transition metals. Calculations performing at this basis set provided acceptable correlations with some experimental results.^40^ The geometrical structures of copper(ii) complex with different spin configurations were examined and the lowest energy one was kept for further analysis. The optimization was followed by a single-point calculation at the M05-2X/6-311++G(2df,2p) model chemistries.

Natural bond orbital (NBO) analyses were also performed in order to provide more insight into electron density transfer between the two ligands and Cu(ii) ion. The extend of these interactions was quantified by means of the second order perturbation energy values (*E*^(2)^) estimated from the following equation:^41^3
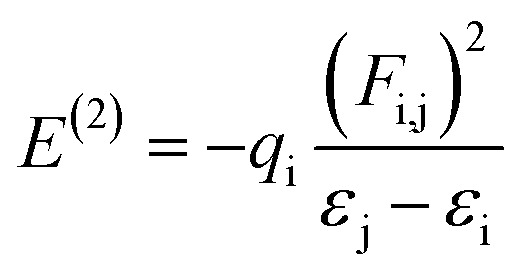
where *ε*_j_ − *ε*_i_ was the energy difference between donor and acceptor i and j NBO orbitals and *F*_i,j_ is the Fock matrix element between i and j NBO orbitals.

Natural population charges for heavy atoms including C, N, O and Cu, electrostatic potential map and frontier orbitals distributions were also analysed for further prediction of local reactivity.

Three common mechanisms including hydrogen atom transfer (HAT), single electron transfer (SET) and proton loss (PL) were considered in this study to analyse the antioxidant potential of the ligands and copper(ii) complex.

• Hydrogen atom transfer (HAT):

R1 Anti − H → (Anti)˙ + H˙, (BDE)

• Single electron transfer (SET):

R2 Anti − H → (Anti − H)˙^+^ + e^−^, (IE)

R3 Anti − H + e^−^ → (Anti − H)˙^−^, (EA)

• Proton loss (PL):

R4 Anti − H → (Anti)^−^ + H^+^, (PA)

The thermochemical properties characterizing the above mechanisms including bond dissociation enthalpy (BDE), adiabatic ionization energy (IE) and electron affinity (EA) and proton affinity (PA) were systematically calculated in the gas phase at 298.15 K and 1 atm.

4 BDE = *H*(Anti˙) + *H*(H˙) − *H*(Anti − H)

5 IE = *H*(Anti − H˙^+^) + *H*(e^−^) − *H*(Anti − H)

6 EA = *H*(Anti − H) + *H*(e^−^) − *H*(Anti − H˙^−^)

7 PA = *H*(Anti)^−^ + *H*(H^+^) − *H*(Anti − H)

where *H* was the total enthalpy of the studied species at 298.15 K and is usually estimated from the following expression:

8 *H* = *E*_0_ + ZPE + *H*_trans_ + *H*_rot_ + *H*_vib_ + *RT*,

*H*_trans_, *H*_rot_ and *H*_vib_ were the translational, rotational, and vibrational contributions to the enthalpy, respectively. *E*_0_ was the total energy at 0 K, and ZPE was the zero-point vibrational energy.

The enthalpy value for the hydrogen atom (H˙) was calculated at the same level of theory. The enthalpies in the gas phase of proton (H^+^) being 0.00236 Hartree (5/2*RT*, the value of an ideal gas) has widely been accepted.^42^ The effect of water on the thermochemical properties was also investigated based on integral equation formalism of the polarizable continuum model (IEF-PCM) at the same level of theory.^43^ Solvation enthalpies of proton and electron in water solvent were calculated using computational approach proposed by Marković *et al.* (2016).^44^ When a proton or an electron is surrounded by the water molecules, it will bind to the water molecule to form a positive or a negative charged particle, H_2_O_sol_^+^ or H_2_O_sol_^−^, respectively. These charged particles are then embedded in a dielectric continuum. This computational approach has been accepted and widely used in several works in the field of antioxidant compounds.^45,46^ As a result, the enthalpies of proton and electron in water were equal to −981.8 and −48.3 kJ mol^−1^, respectively.

## 4 Results and discussion

### 4.1. Synthesis and characterization

**Scheme 1:**
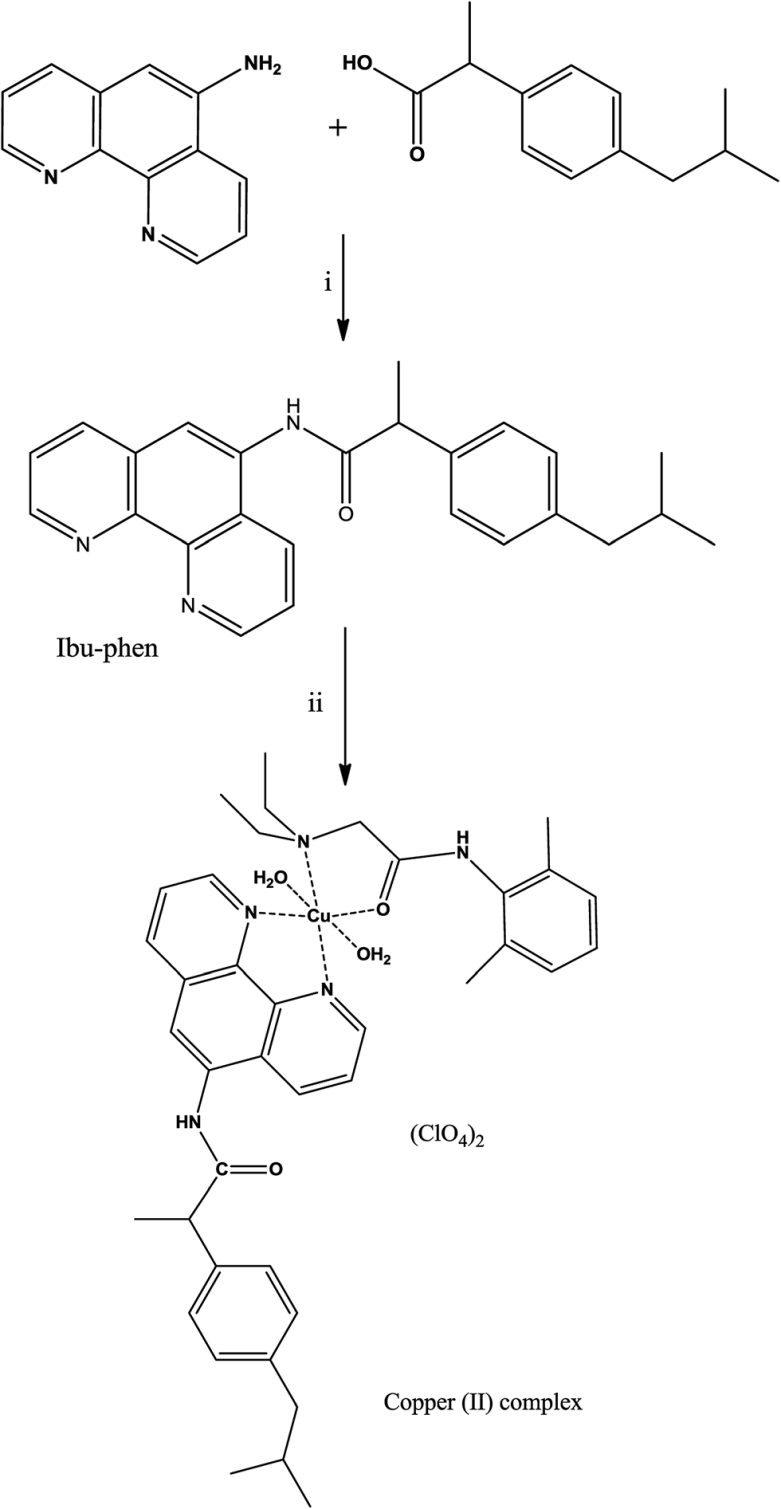
Synthetic pathways of the ligand Ibu-phen and the copper(ii) complex. Reagents and conditions: (i) EDC, HOBt, DIEA, DMF, 0 °C to rt., 15 h, 86%; (ii) Cu(ClO_4_)_2_·6H_2_O, CH_3_CN : CH_2_Cl_2_ 1 : 1, 50 °C, 24 h, 72%.

The ligand ibuprofen amide-phenanthroline (Ibu-phen) was synthetized using a slightly modified literature method.^30^ Coupling of ibuprofen with 1,10-phenanthrolin-5-amine was carried out using *N*-(3-dimethylaminopropyl)-*N*′-ethylcarbodiimide hydrochloride (EDC-HCl), *N*,*N*-diisopropylethylamine (DIEA) and 1-hydroxybenzotriazole hydrate (HOBt·*x*H_2_O) in DMF *via* the procedure outlined in the Experimental section (Scheme 1). Good yield and pure ligand of Ibu-phen are obtained when the reaction is run at a temperature range of about 0 °C to room temperature. The ligand Ibu-phen is highly soluble in methanol but insoluble in other solvents such as water, benzene, toluene, acetone, ether, acetonitrile, dichloromethane and chloroform. The success of the coupling reaction is demonstrated in the ^1^H NMR spectrum by the disappearance of the signals of amino proton of the starting 1,10-phenanthrolin-5-amine at 6.16 ppm and of COOH proton of ibuprofen at 12.21 ppm (Fig. S1 and S2 of the ESI†).^47,48^

The copper(ii) complex was obtained by reacting Cu(ClO_4_)_2_·6H_2_O with ibuprofen amide-phenanthroline (Ibu-phen) and lidocaine (LC) in acetonitrile/dichloromethane (1 : 1) at 50 °C (Scheme 1). The copper(ii) complex is soluble in methanol, DMF, DMSO, and water but insoluble in other organic solvents. The mass spectrum of ligand Ibu-phen shows peaks of [M + H]^+^ centered at *m*/*z* 384.2076 and 385.1132 while in the spectrum of copper complex we observed major isotopes of the fragment of [M − (ClO_4_)]^+^ at *m*/*z* 815.2722 and 817.2345 (Fig. S3 and S4 of the ESI†). In the IR spectrum of copper complex, the *ν*(C=O) bands were shifted to lower frequencies at 1648 cm^−1^ (1662 cm ^−1^ in LC) due to the coordination of oxygen atom to the copper ion.^24^ The copper(ii) complex shows broad *ν*(H_2_O) band at 3356 cm^−1^ in coherence with the presence of coordinated water (Fig. S5–S7 of the ESI†).^49^

### 4.2. UV-Vis spectroscopy studies and stability in solution

The UV-Vis spectrum of the copper(ii) complex in the solid state (Fig. S8†) showed two bands at 408 nm and 702 nm. The broadness of the band at 702 nm showed the three transitions ^2^B_1g_ → ^2^A_1g_ (*ν*_1_), ^2^B_1g_ → ^2^B_2g_ (*ν*_2_) and ^2^B_1g_ → ^2^E_g_ (*ν*_3_), due to dynamic Jahn-Teller distortion and suggested the distorted octahedral geometry (*D*_4h_) around Cu(ii) center.^50^ In the DMSO solution, the absorbance bands were observed at the same wavelength, indicating that the copper(ii) complex is structurally stable in DMSO solution and no significant change in the coordination environment of the Cu(ii) ion was observed.

In addition, the stability of copper(ii) complex was monitored over 72 h in DMSO solution at room temperature (Fig. S9†). The results did not show any significant change in either the intensity or the position of the absorption bands, which confirmed the stability of the copper(ii) complex in DMSO solution.

### 4.3. Electron paramagnetic resonance (EPR) spectroscopy of copper(ii) complex

**Fig. 1:**
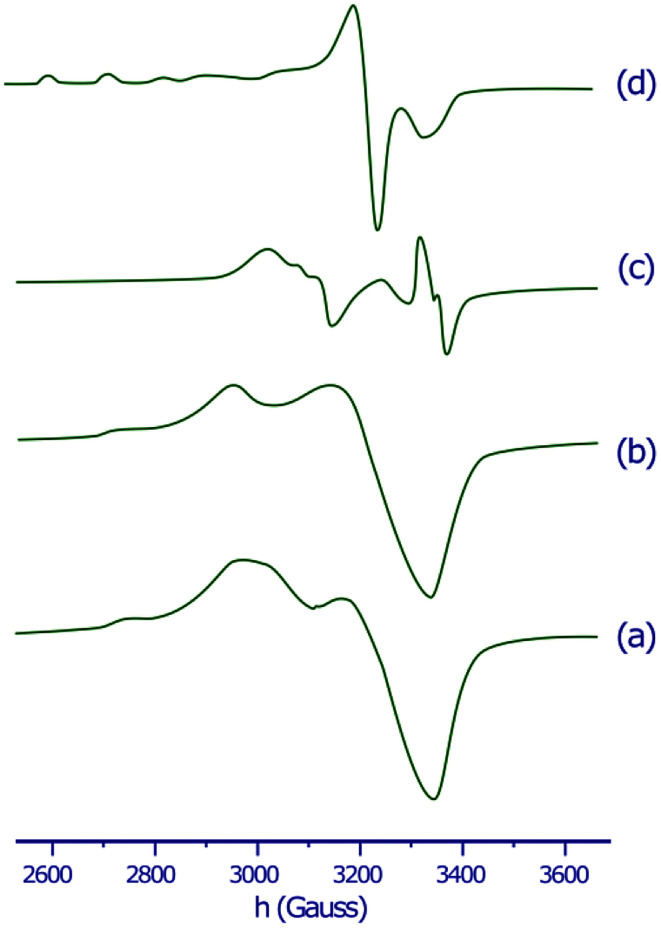
EPR spectra of the copper(ii) complex in the solid state at 298 K (a), in the solid state at 77 K (b), in DMSO solution at 298 K (c) and in DMSO solution at 77 K (d).

The X-band EPR spectra of the copper(ii) complex in the solid state and in DMSO solution were recorded at both 298 K and 77 K to clarify the coordination environment around Cu(ii) center. The EPR spectra of the copper(ii) complex are displayed in Fig. 1. The spectral parameters and their assignments are presented in Table 1.

**Table 1:** EPR spectral parameters of the copper(ii) complex

Copper(ii) complex	Solid (298 K)	Solid (77 K)	DMSO (298 K)	DMSO (77 K)
*g*_∥_	2.374	2.371	2.412	2.403
*g*_⊥_	2.092	2.088	2.098	2.095
a *A*_∥_	148	142	149	141
b *G*	4.144	4.302	4.281	4.322

a *A*_∥_ values have 10^−4^ cm^−1^ units.

b *G* = (*g*_∥_ − 2.0023)/(*g*_⊥_ − 2.0023).

The EPR spectra for both the solid sample and in DMSO solution have the same features. The EPR spectrum of the copper(ii) complex both at 298 K or 77 K as solid or in DMSO solution, shows the distorted octahedral symmetry center, for which the evaluated parameters are: *g*_∥_ > *g*_⊥_, and *A*_∥_ = 141 − 149 × 10^−4^ cm^−1^. The obtained *A*_∥_ value for copper(ii) complex is comparable with *A*_∥_ found for six-coordinate complexes of distorted octahedral geometries, such as the bis(pyridine-2,6-diimine) Cu(ii) complex (*A*_∥_ = 145 × 10^−4^ cm^−1^), or the [Cu(NOTA)]^−^ complex (NOTA = 1,4,7-triazacyclononane-1,4,7-triacetate) (*A*_∥_ = 149.5 × 10^−4^ cm^−1^).^51,52^ The *g*_∥_ > *g*_⊥_ > 2.0023 observed for the copper(ii) complex is consistent with a copper(ii) (d^9^) ion in axial symmetry and the unpaired electron in the d_*x*^2^−*y*^2^_ orbital.^53^ It was reported that *g*_∥_ values vary in the ranges of 2.3–2.4 and 2.2–2.3 for complexes owning Cu–O and Cu–N bonds, respectively.^54–57^ Therefore, *g*_∥_ values 2.37–2.41 found for our copper(ii) complex are in agreement with both Cu–O and Cu–N bonds in this complex. A minor variation in the point symmetry from octahedral geometry is observed for mixed copper–nitrogen and copper–oxygen system.^57^ The calculated geometric parameter *G* using the equation *G* = (*g*_∥_ − 2.0023)/(*g*_⊥_ − 2.0023) for the copper(ii) complex is higher than 4, this predicts that the exchange interaction between the metal centers is minor.^58^

### 4.4. Thermal analysis

**Fig. 2:**
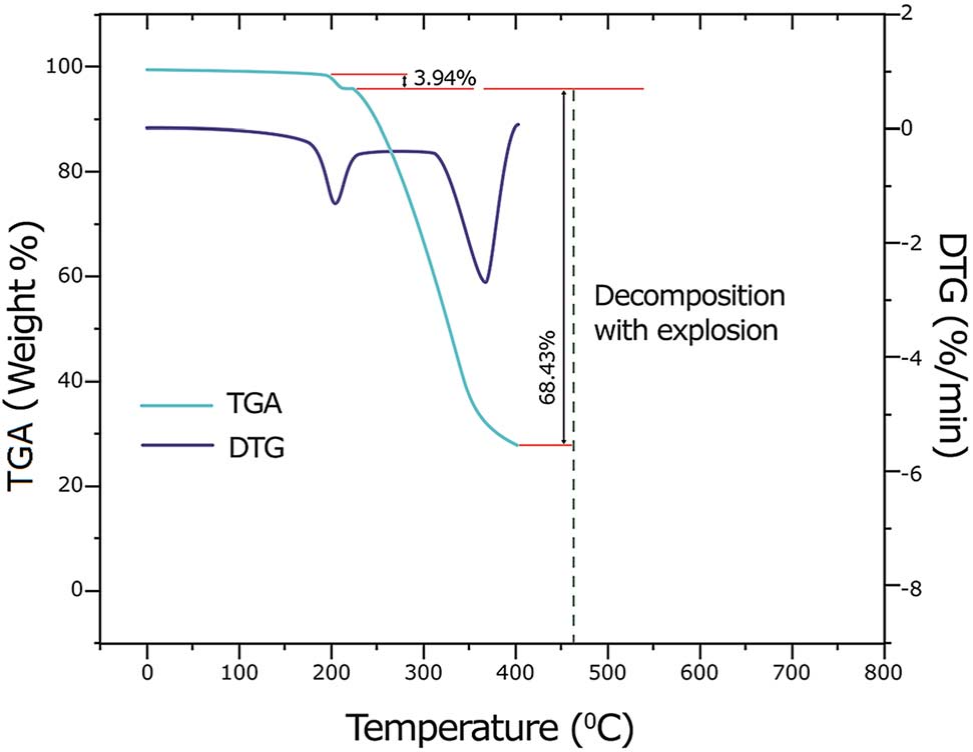
TGA and DTG curves for copper(ii) complex.

By using thermogravimetric analysis (TGA), we can get information whether the water molecules are inside or outside the inner coordination sphere of the central metal ion. The temperature range at 90–140 °C corresponds to the weak binding of water and region 150–220 °C corresponds to the strong binding of water (or coordinated water molecules).^59^ Analysis of the TGA and differential thermogravimetric analysis (DTG) curves of the copper(ii) complex (Fig. 2) indicates a weight loss of 3.94% (calcd 3.93%) at a temperature range 178–218 °C (DTG_max_ = 201 °C) which corresponds to the removal of two coordinate water molecules. A weight loss of 68.43% (calcd 68.40%) is observed in the temperature range 218–402 °C (DTG_max_ = 364 °C) which suggests the elimination of two ligands LC and Ibu-phen molecules. Further heating causes to decompose with explosion at 462 °C (735.15 K). The explosion of the complexes containing perchlorate anion during the thermal decomposition was observed for other compounds such as [Ca(NH_3_)_6_](ClO_4_)_2_, [Sr(OS(CH_3_)_2_)_6_](ClO_4_)_2_, [Mn(NH_3_)_6_](ClO_4_)_2_, and [Ca(H_2_O)_4_](ClO_4_)_2_ that the temperature of explosion changed from 540 K to above 700 K.^60–62^

### 4.5. Antioxidant activity

Oxidative stress results from an imbalance between two processes, one leading to reactive radical production and the other removing these species. Although these free radicals are formed during normal cellular functions in the body, their excess amount may play a crucial role in developing various diseases including cancer and chronic inflammation. Antioxidants can defense human health from reactive radicals and decrease oxidative stress.

**Fig. 3:**
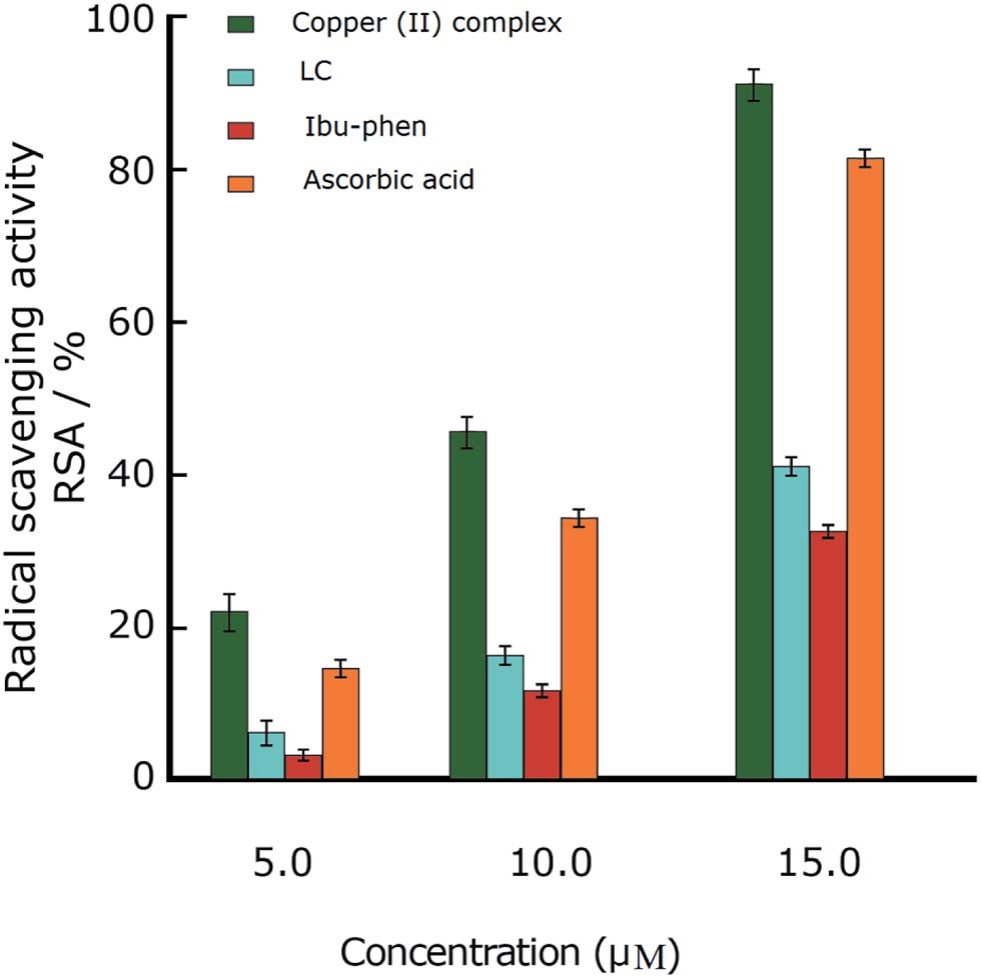
Radical scavenging activity (RSA%) of DPPH˙. Comparison of different concentrations of LC, Ibu-phen, copper(ii) complex and ascorbic acid. Data are shown as means ± SEM (*n* = 3).

**Fig. 4:**
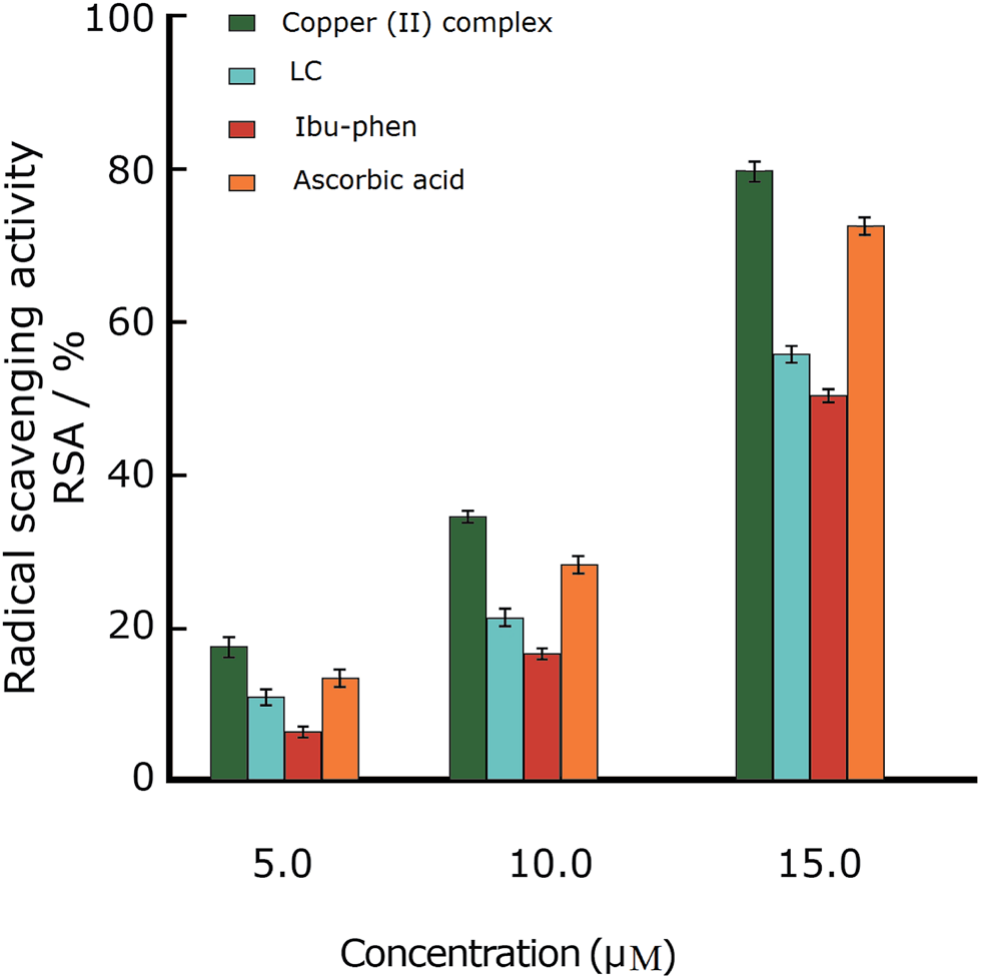
Radical scavenging activity (RSA%) of ABTS˙^+^. Comparison of different concentrations of LC, Ibu-phen, copper(ii) complex and ascorbic acid. Data are shown as means ± SEM (*n* = 3).

**Fig. 5:**
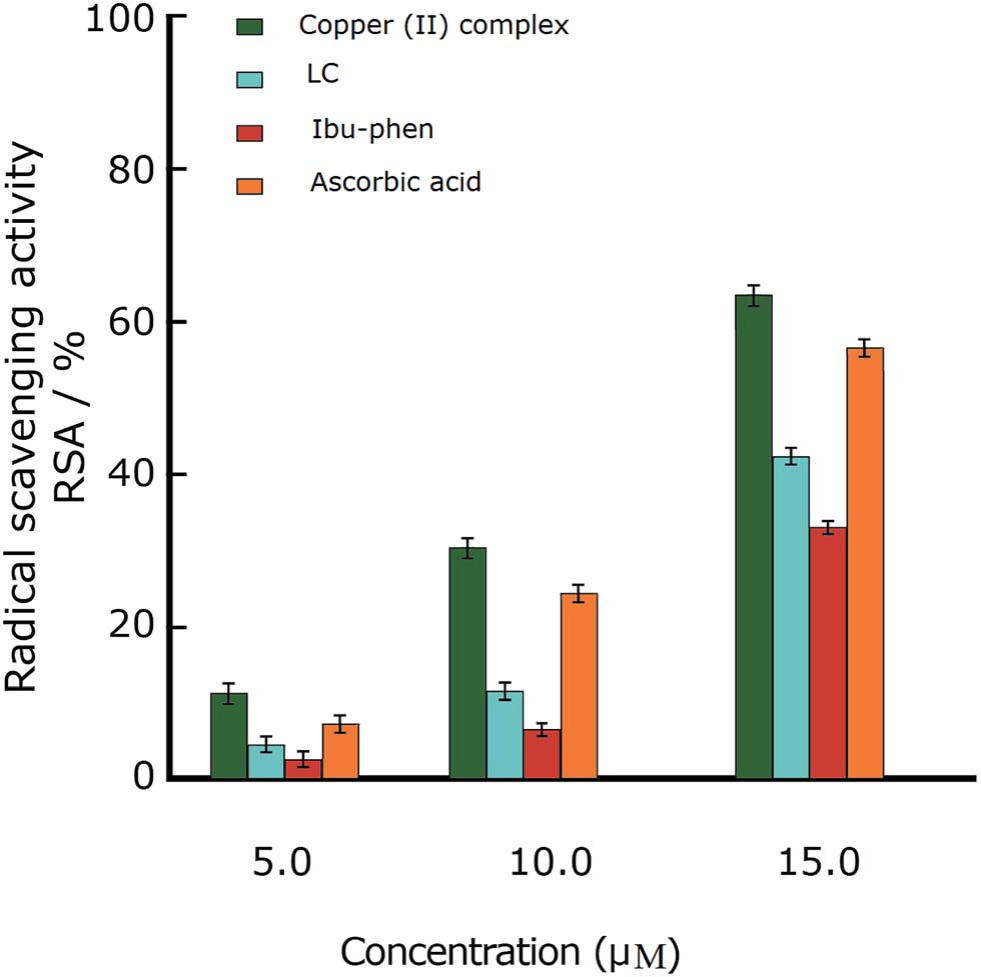
Radical scavenging activity (RSA%) of OH˙. Comparison of different concentrations of LC, Ibu-phen, copper(ii) complex and ascorbic acid. Data are shown as means ± SEM (*n* = 3).

In this part we discuss the antioxidant activity of free ligands and copper(ii) complex evaluated in a series of *in vitro* assay involving HO˙ radicals, DPPH˙ radicals and ABTS˙^+^ cation radicals.^63–65^ The results are shown in the Fig. 3–5. The IC_50_ values of all samples including two ligands, copper(ii) complex and ascorbic acid as a reference are summarized in the Table 2.

**Table 2:** *In vitro* antioxidant activity of the free ligands and copper(ii) complex

Samples	Radical scavenging activities^a^ (IC_50_, μM)		
	DPPH˙	ABTS˙^+^	HO˙
LC	59.20 ± 0.10	24.20 ± 0.20	54.30 ± 0.10
Ibu-phen	63.20 ± 0.30	27.30 ± 0.10	57.60 ± 0.18
Copper(ii) complex	1.21 ± 0.21	2.92 ± 0.10	4.05 ± 0.05
Ascorbic acid	3.24 ± 0.10	3.84 ± 0.15	5.17 ± 0.20

a IC_50_ (50% concentration of inhibition for radical scavenging activity). Values are mean ± SD of triplicates.

#### 4.5.1. DPPH˙ radical

The model of the scavenging of DPPH˙ radicals is simple, rapid and this is considered as an appropriate method to study the antioxidant property of compounds. The DPPH˙ radicals are generally stable except in the presence of compounds capable of donating hydrogen atoms, in which the radical sweep results a color change from purple to yellow.^64^

In the DPPH˙ assay (0.1 mM), the IC_50_ (50% concentration of inhibition for radical scavenging activity) values for LC, Ibu-phen and copper(ii) complex were found as 59.20, 63.20 and 1.21 μM, respectively. Under our reaction conditions IC_50_ value of ascorbic acid was 3.24 μM (Table 2). Free radical scavenging activity (RSA) at various concentrations was decreased in the following order: copper(ii) complex > ascorbic acid > LC > Ibu-phen (Fig. 3).

#### 4.5.2. ABTS˙^+^ radical

2,2′-Azino-bis-(3-ethylbenzothiazoline-6-sulphonic acid) (ABTS˙^+^) is also a free and stable radical cation. This radical is reactive towards most antioxidants such as phenols, thiols or compounds that can give a hydrogen atom or an electron. The ABTS˙^+^ test is usually used to evaluate the antioxidant capacity of the biological fluids and many pure compounds.

Under these conditions, the blue ABTS˙^+^ radical cation becomes colorless on reduction. This radical cation absorbs light at 734 nm.^17^ In the ABTS˙^+^ assay (1 mM), the IC_50_ values for LC, Ibu-phen, and Cu complex were found as 24.20, 27.30 and 2.92 μM, respectively. Under our reaction conditions, IC_50_ value of ascorbic acid is 3.84 μM (Table 2). Thus, the RSA values at various concentrations were decreased in the following order: copper(ii) complex > ascorbic acid > LC > Ibu-phen (Fig. 4).

#### 4.5.3. HO˙ radical

Hydroxyl radical (HO˙) can damage virtually all types of macromolecules such as carbohydrates, nucleic acids, lipids and amino acids. Because of very short *in vivo* half-life (approximately 10^−9^ seconds), hydroxyl radical is very dangerous species to the organism compared with other free radicals. It attacks proteins, DNA, polyunsaturated fatty acid in membranes and most biological molecules. Therefore, the scavenging of this radical is one of the major aims of antioxidant administration. Hydroxyl radical is known to be capable of abstracting hydrogen atoms from membrane lipids and brings about peroxide reaction of lipids.^64^ The IC_50_ values of HO˙ radical scavenging assay (1 mM) for LC, Ibu-phen, and Cu complex were found as 54.30, 57.60 and 4.05 μM respectively. Under the same conditions, IC_50_ value of ascorbic acid was 5.17 μM (Table 2). Thus, the RSA at various concentrations was decreased in the same order as for the DPPH˙ and ABTS˙^+^: copper(ii) complex > ascorbic acid > LC > Ibu-phen (Fig. 5).

Overall, the antioxidant activity of the free ligands and copper(ii) complex against the free radicals including DPPH˙, ABTS˙^+^ and HO˙ showed that the copper(ii) complex displays greater scavenging activity than the free ligands. Moreover, the copper(ii) complex shows much better scavenging activity than the standard antioxidant, ascorbic acid. Furthermore, the antioxidant activity of the copper(ii) complex against the DPPH˙ radical is better than ABTS˙^+^ and OH˙ radicals and the trend is decreased in the following order: DPPH˙ > ABTS˙^+^ > HO˙. The low IC_50_ values obtained in the antioxidant assays confirm that the copper(ii) complex could be a considerable example for the design of radical-quenching-based drugs.

The antioxidant activities of this copper complex against the DPPH˙ and ABTS˙^+^ radicals are comparable with other copper complexes based on mixed 1,10-phenanthroline and coumarine ligands.^66^ The potent antioxidant activity of this copper(ii) complex is probably related to the use of the ibuprofen amide-phenanthroline ligand, known as antioxidant agents, and the coordination of these ligands to the copper center.

### 4.6. Electron paramagnetic resonance (EPR) analysis for DPPH˙, ABTS˙^+^ and HO˙ radicals scavenging assay

**Fig. 6:**
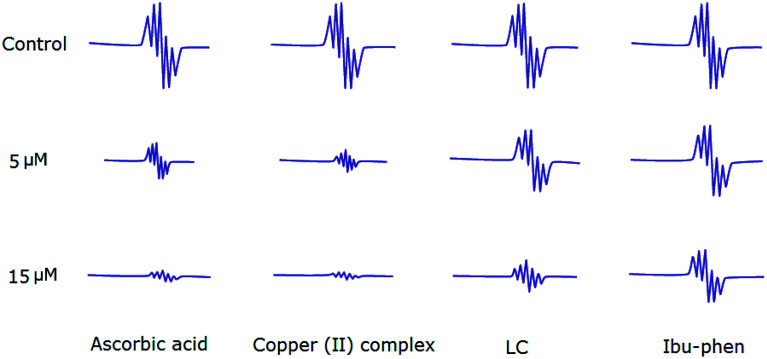
EPR spectra of DPPH˙ radical in the absence and presence (top) of LC, Ibu-phen, copper(ii) complex and ascorbic acid at 5 (middle) and 15 μM (bottom) concentrations.

Antioxidant activities of LC, Ibu-phen, copper(ii) complex, and ascorbic acid were also tested for free radical sources by EPR spin trapping technique. During the addition of copper complex, the EPR signal is decreased as function of concentration, and scavenging pattern is dose-dependent. The copper complex and ascorbic acid scavenge DPPH˙ radical over about 91% and 89% at 15 μM, respectively, and the activity is decreased in the following order: copper(ii) complex > ascorbic acid > LC > Ibu-phen (Fig. 6).

**Fig. 7:**
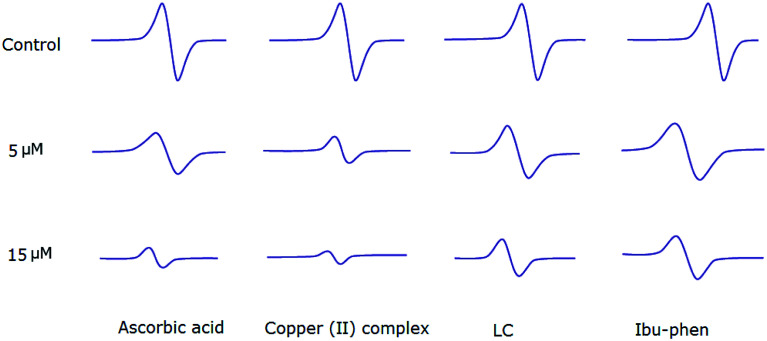
EPR spectra of ABTS˙^+^ radical in the absence and presence (top) of LC, Ibu-phen, copper(ii) complex and ascorbic acid at 5 (middle) and 15 μM (bottom) concentrations.

Copper(ii) complex also quenches ABTS˙^+^ radical and the scavenging ratio is 77% at 15 μM that is comparable with ascorbic acid, *i.e.* 69%, at the same concentration (Fig. 7).

**Fig. 8:**
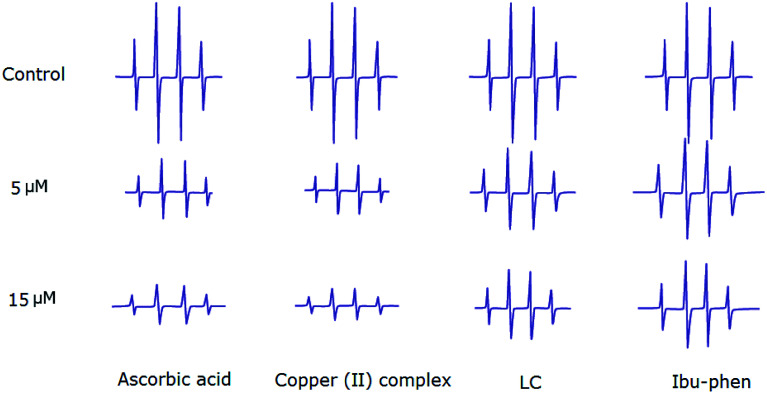
EPR spectra of HO˙ radical in the absence and presence (top) of LC, Ibu-phen, copper(ii) complex and ascorbic acid at 5 (middle) and 15 μM (bottom) concentrations.

Hydroxyl radicals generated in Fe^2+^/H_2_O_2_ system are trapped by DMPO forming spin adduct which can be detected by an EPR spectrometer. The typical 1 : 2 : 2 : 1 EPR signal of the DMPO–OH adduct is clearly observed (Fig. 8). The ESR results show that copper(ii) complex and ascorbic acid suppress about 66% and 62% of the hydroxyl radical at 15 μM, respectively. Antioxidant activity decreases in the following order: copper(ii) complex > ascorbic acid > LC > Ibu-phen (Fig. 8).

EPR studies indicate that copper complex is the most potent scavenger of the DPPH˙, ABTS˙^+^ and HO˙ radicals compared with ascorbic acid and free ligands. The scavenging activities of the DPPH˙, ABTS˙^+^ and HO˙ radicals obtained by EPR spectroscopy are comparable with the results of UV-Vis study.

### 4.7. Mechanism of DPPH˙ radical scavenging assay by copper complex

**Fig. 9:**
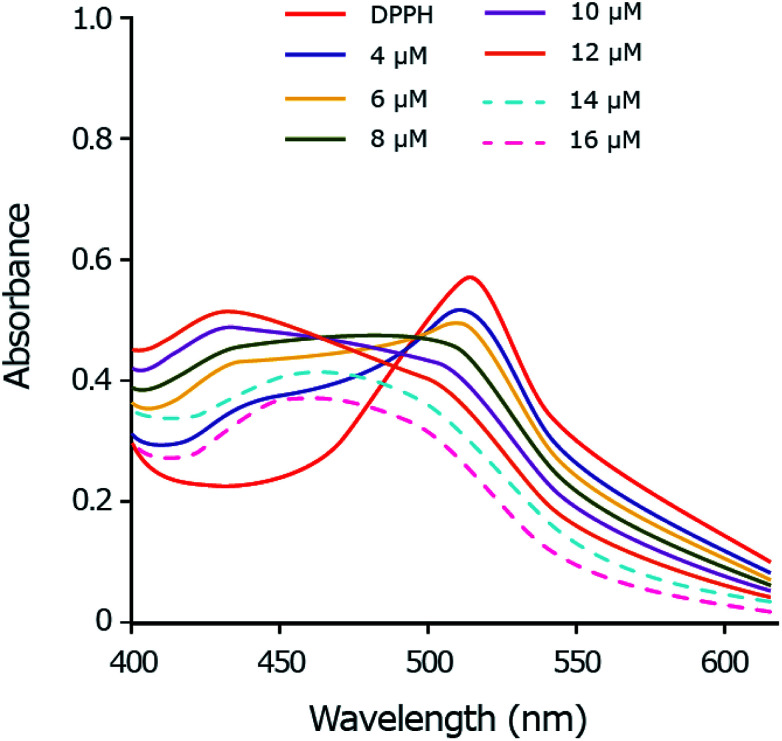
UV-Vis spectral monitoring of reaction between DPPH˙ (60 μM) and copper complex (4–16 μM) in methanol: DMSO (99.5 : 0.5).

As the copper complex shows stronger radical scavenging activity towards DPPH˙ than towards ABTS˙^+^ or HO˙ radical, the scavenging mechanism of DPPH˙ by copper(ii) complex is chosen to be studied in details. The DPPH˙ is characterized by an absorbance at 514 nm by UV-Vis. By adding the copper complex, the concentration of DPPH˙ decreases following by the decrease of visible absorption band at 514 nm. Meanwhile, by accepting one electron from copper(ii) complex, the DPPH^−^ anion is formed and shows the absorption peak around 431 nm.^67,68^ The intensity of the band at 431 nm increases as a function of the copper complex concentration up to 12 μM which demonstrates the anion form of DPPH (Fig. 9). In the excess amount of copper(ii) complex (14 to 16 μM), the band at 431 nm is disappeared due to the exchange of the anion form of DPPH^−^ to DPPH–H. The DPPH–H is formed by accepting one proton from the copper complex.^68,69^

This study suggests a two-step mechanism for the scavenging reaction of DPPH˙ by copper(ii) complex including the electron-accepting in the first and the proton-accepting in the second step.

### 4.8. Computational study of antioxidant activity

#### 4.8.1. Optimized structures and electronic properties

**Fig. 10:**
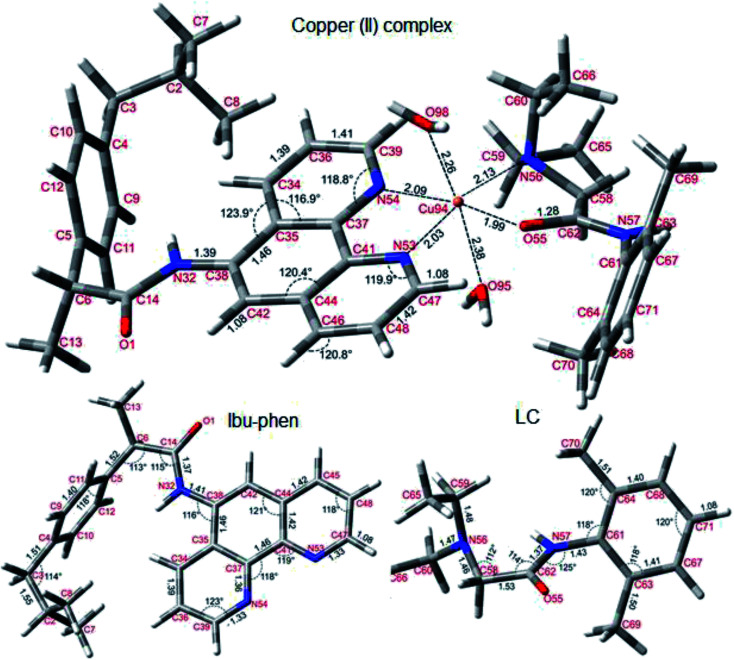
Optimized structures of copper(ii) complex, Ibu-phen and LC ligands at the M05-2X/LanL2DZ level of theory.

Optimized geometries of copper(ii) complex, Ibu-phen and LC investigated at the M05-2X/LanL2DZ level of theory are shown in Fig. 10. The Cu(ii) ion is coordinated with the ligands by distorted octahedral geometry in which both Ibu-phen and LC act as bidentate ligands and two water molecules. The copper(ii) complex structure wasoptimized using different spin multiplicities from 2, 6 and 8 to evaluate influence of its spin states on stabilization of the complex. As a result, the structure with doublet states (multiplicity being 2) was obtained with the lowest energy. Cartesians coordinates and molecular enthalpies of copper(ii) complex with three multiplicity values are resumed in Table S1 of the ESI.†

As can be seen in Fig. 10, Ibu-phen is coordinated to Cu(ii) ion through its two nitrogen atoms, *i.e.* N53 and N54, while LC binds with the central cation *via* N56 and O55 atoms. The atomic distances of N53–Cu94 and N54–Cu94 are equal to 2.03 and 2.09 Å, while the atomic distances of N56–Cu94 and O55–Cu94 are 1.99 and 2.13 Å, respectively. Moreover, the Cu(ii) ion coordinates also with two water molecules with the distances of O98–Cu94 and O95–Cu94 equal to 2.26 and 2.38 Å.

**Fig. 11:**
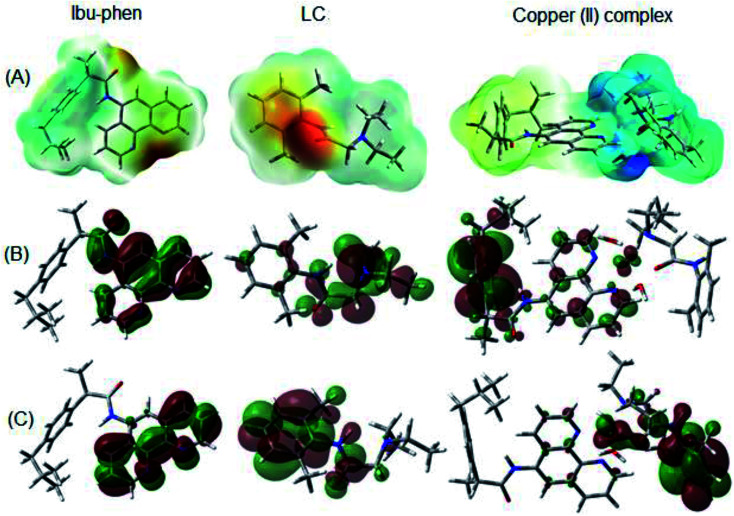
(A) ESP maps, (B) HOMO and (C) LUMO distributions of Ibu-phen, LC ligands and copper(ii) complex.

Molecular electrostatic potential consists in an efficient approach to find the reactive sites of a molecule for electrophilic and nucleophilic attacks.^70,71^ The reactive site consists in partially charged regions of a molecule that have affinities for interacting with charged particles. The ESP maps of Ibu-phen, LC and copper(ii) complex are displayed in Fig. 11A.

As can be observed in Fig. 11A, the most negative regions expressed in red color are located at the heteroatoms of the ligands, such as N53, N54 and O1 on Ibu-phen, or O55, N56 and N57 on lidocaine. This observation is also confirmed by natural population analysis (NPA) charges resumed in Table S2 of the ESI.†

The highest occupied molecular orbital (HOMO) and the lowest unoccupied molecular orbital (LUMO) are two of the most important frontier orbitals which characterize the local reactivity of the studied compounds.^72^ The shapes of HOMO and LUMO of Ibu-phen, LC and the copper(ii) complex are shown in Fig. 11B and C.

As observed in Fig. 11, the HOMO and LUMO for Ibu-phen are all distributed across the phenanthroline moiety, while for LC the HOMO is principally delocalized across the diethyl-amino substituent and its LUMO is concentrated at the phenyl ring. In case of the copper(ii) complex, the HOMO is mainly localized at the Ibu-phen ligand and while its LUMO is distributed at the LC one. Moreover, the delocalized regions of the complex are essentially found at the phenyl moieties of the two ligands.

#### 4.8.2. Natural bond orbital (NBO) analysis

The natural bond orbital of the copper(ii) complex is also analyzed and the obtained results are presented in Table S3 of the ESI.† Generally, the Cu94 cation plays as an electron acceptor in coordinating with the two ligands and two water molecules.

In fact, electron density is essentially transferred from the first lone pair of electron on N53 atom, LP(1) N53, of Ibu-phen to the unoccupied orbitals on copper such as LP*(5) Cu94, LP*(6) Cu94 and LP*(8) Cu94 with lower stabilization energies of 120.2, 49.6 and 52.7 kJ mol^−1^, respectively. Similarly, the electron density is also donated from the first lone pair of electron from N54 atom, LP(1) N54, to the vacant orbitals on copper with lower stabilization energies from 37.1 to 98.9 kJ mol^−1^.

Regardless of the electron transfer from LC ligand, the electrons are donated from the σ orbital on the N56–C59 bond, σ(1) N56–C59, to the vacant orbital on Cu cation, LP*(9) Cu94, with a stabilization energy of 237.1 kJ mol^−1^. The first electron lone pairs located on N56 atom, LP(1) N56, are transferred to unoccupied orbitals on Cu94 cation, LP*(5) Cu94, LP*(6) Cu94 and LP*(8) Cu94, with energy values from 35.9 to 73.8 kJ mol^−1^. Moreover, the electrons are also donated from the lone pairs of electrons on O55 atom of LC, LP(1) O55 and LP(2) O55 and LP(3) O55, to the vacant orbitals on the Cu94 ion with lower stabilization energies from 26.2 to 395.8 kJ mol^−1^.

Two H_2_O molecules which are attached to the central Cu(ii) ion also contribute their lone pairs of electrons to the vacant orbitals of the metal. Indeed, the second lone pairs of electrons on O95 atom, *i.e.* LP(2) O95, and O98 one, *i.e.* LP(2) O98, are donated to LP*(9) Cu94 with lower stabilization energies of 81.3 and 844.9 kJ mol^−1^, respectively. Thus, the existence of two water molecules improves significantly the stabilization of the copper(ii) complex.

### 4.9. Characteristic thermochemical properties for antioxidant activity

#### 4.9.1. Hydrogen transfer (HT) mechanism

Generally, antioxidant capacity of a compound *via* HT mechanism is characterized by its bond dissociation enthalpy (BDE) property (reaction (R1) and eqn (2)), the lower BDE value the higher antioxidant capacity *via* HT mechanism.

The lowest BDE values for Ibu-phen, LC and copper(ii) complex calculated in the gas phase and in water solvent at the M05-2X/6-311++g(2df,2p)//M05-2X/LanL2DZ level of theory are resumed in Table 3.

**Table 3:** The lowest BDE values (kJ mol^−1^) for Ibu-phen, LC and copper(ii) complex calculated at the M05-2X/6-311++g(2df,2p)//M05-2X/LanL2DZ level of theory^a^

Bond position	Bond dissociation enthalpy, BDE (kJ mol^−1^)		
	Ibu-phen	LC	Copper(ii) complex
Ibu-C2–H	396.2		
Ibu-C3–H	376.3		
Ibu-C6–H	**358.7 (360.0)**		701.3 (459.8)
LC-C58–H		**354.6 (339.6)**	686.9 (350.5)
LC-C59–H		366.9	
LC-C60–H		365.5 (364.0)	**596.3 (305.6)**

a Values in parentheses correspond to BDE values obtained in water solvent.

For Ibu-phen, the C6–H bond located beside the O1 heteroatom has the lowest BDE, *i.e.* 358.7 kJ mol^−1^ in the gas phase. While for LC ligand the easiest H-atom donating site is found at the C58–H bond with BDE value being 354.6 kJ mol^−1^ in the gas phase. The corresponding BDE values of the copper(ii) complex for the same positions on the two ligands are also calculated. As a result, BDE values are equal to 701.3 and 686.9 kJ mol^−1^ for the C6–H and C58–H bonds, respectively. Moreover, the lowest BDE value of copper(ii) complex is found at the C60–H position with value being 596.3 kJ mol^−1^.

The same trend of activity is observed in water solvent with considerable decrease of all BDE values. For example, BDEs obtained in water at the C6–H position for Ibu-phen and copper(ii) complex increase from 360.0 to 459.8 kJ mol^−1^, respectively. However, an inverse observation is noted for BDEs obtained in water for C60–H position which are equal to 364.0 and 305.6 kJ mol^−1^ for LC ligand and copper(ii) complex, respectively.

Thus, the calculated data for C60–H position is in good agreement with the experimental one obtained from DPPH˙ essay which indicates that the radical scavenging activity of copper(ii) complex in the solvent is always higher than LC and Ibu-phen (Fig. 2).

#### 4.9.2. Single electron transfer (SET) mechanism

Table 4 represents ionization energy (IE) and electron affinity (EA) values obtained for Ibu-phen, LC and copper(ii) complex calculated in the gas phase and in water solvent (reactions (R2), (R3) and equations eqn (5) and (6)) at the M05-2X/6-311++g(2df,2p)//M05-2X/LanL2DZ level of theory.

**Table 4:** IE and EA (kJ mol^−1^) for Ibu-phen, LC and copper(ii) complex calculated at the M05-2X/6-311++g(2df,2p)//M05-2X/LanL2DZ level of theory^a^

	Ibu-phen	LC	Copper(ii) complex
IE (eV)	723.6 (551.3)	719.8 (500.7)	1105.7 (647.3)
EA (eV)	56.0 (188.4)	88.8 (52.8)	661.9 (278.6)

a Values in parentheses correspond to the results obtained in water solvent.

As a result, IE and EA values in the gas phase for Ibu-phen are equal to 723.6 and 56.0 kJ mol^−1^, respectively. Meanwhile, these values for LC ligand are 719.8 and 88.8 kJ mol^−1^, respectively. The copper(ii) complex has an increased IE value in the gas phase, *i.e.* 1105.7 kJ mol^−1^, being 1.5-fold higher than the one of two ligands, while its EA value, *i.e.* 661.9 kJ mol^−1^, is 7- to 11-fold higher than the one of LC and Ibu-phen, respectively. The considerably higher EA value of copper(ii) complex by compared with the ones of two ligands indicates its high antioxidant activity *via* the electron-accepting action. Thus, the antioxidant potential of the compounds can be classified in descending order: copper(ii) complex > LC > Ibu-phen. This order is quite consistent with the results observed from ABTS˙^+^ essay (Fig. 3). The same trend is also observed in water solvent with the higher EA value of copper(ii) complex, *i.e.* 278.6 kJ mol^−1^, by compared with the values of Ibu-phene and LC, *i.e*. 188.4 and 52.8 kJ mol^−1^, respectively.

Adiabatic reaction enthalpy (Δ*H*) and Gibbs free energy (Δ*G*) of SET reaction between the two ligands and copper(ii) complex with different free radicals are also investigated. The electron-donating/-accepting reactions between the potential antioxidant (Anti) and free radicals (R˙) may occur as follows:^65^

R5 Anti + (R˙) → (Anti˙^+^) + (R^−^)

R6 Anti + (R˙) → (Anti˙^−^) + (R^+^)

For reaction (R5), the adiabatic Δ*H*^0^ and Δ*G*^0^ values are calculated as follows:

9 Δ*H*^0^_donor_ = [H(Anti˙^+^) + H(R^−^)] − [H(Anti) + H(R˙)]

10 Δ*G*^0^_donor_ = [G(Anti˙^+^) + G(R^−^)] − [G(Anti) + G(R˙)]

Whereas for reaction (R6), the calculations are as follows:

11 Δ*H*^0^_acceptor_ = [H(Anti˙^−^) + H(R^+^)] − [H(Anti) + H(R˙)]

12 Δ*G*^0^_acceptor_ = [G(Anti˙^−^) + G(R^+^)] − [G(Anti) + G(R˙)]

The free radicals considered for the above reactions include HOO˙, CH_3_OO˙, HO˙, ABTS˙^+^ and DPPH˙. The HOO˙ and CH_3_OO˙ are chosen, because they are the simplest members of the peroxyl radicals (ROO˙) family which represent moderate reactivity and are suggested to be used for accurate prediction of rate constants for free radical scavenging processes.^73^ Whereas the HO˙, ABTS˙^+^ and DPPH˙ radicals consist in the ones used in the experimental antioxidant capacity essays.

The reaction enthalpies (Δ*H*^0^) and Gibbs free energies (Δ*G*^0^) (in kJ mol^−1^, at 298.15 K) for the electron-donating/-accepting reactions ((R5) and (R6)) of the studied compounds and the free radicals calculated in the gas phase and in water solvent are all resumed in Tables 5 and 6, respectively.

**Table 5:** Reaction enthalpies (Δ*H*^0^, kJ mol^−1^) at 298.15 K for the reactions (R5) and (R6) between different radicals (R˙) and the potential antioxidants (Anti) in the gas phase, calculated at the M05-2X/6-311++g(2df,2p)//M05-2X/LanL2DZ level of theory^a^

Free radical	(R5): Anti + (R˙) → (Anti˙^+^) + (R^−^)			(R6): Anti + (R˙) → (Anti˙^−^) + (R^+^)		
	Ibu-phen	LC	Copper(ii) complex	Ibu-phen	LC	Copper(ii) complex
HOO˙	632.2 (200.0)	627.9 (149.3)	1003.4 (296.0)	1117.8 (632.3)	1262.8 (767.9)	511.9 (542.1)
CH_3_OO˙	623.1 (206.7)	618.8 (156.1)	994.3 (302.7)	995.3 (559.2)	1140.3 (694.8)	389.4 (469.0)
HO˙	565.9 (104.9)	561.7 (54.2)	937.2 (200.9)	1497.3 (977.8)	1642.3 (1113.4)	891.4 (887.6)
ABTS˙^+^	54.3 (168.1)	50.1 (117.4)	425.6 (264.1)	959.2 (443.9)	1104.1 (579.5)	353.2 (353.7)
DPPH˙	389.4 (129.2)	385.1 (78.5)	760.6 (225.2)	632.3 (301.4)	777.3 (437.0)	26.4 (211.2)

a Values in parentheses correspond to the results obtained in water solvent.

**Table 6:** Gibbs free energy (Δ*G*^0^, kJ mol^−1^) at 298.15 K for the reactions (R5) and (R6) between different radicals (R˙) and the potential antioxidants (Anti) in the gas phase, calculated at the M05-2X/6-311++g(2df,2p)//M05-2X/LanL2DZ level of theory^a^

Free radical	(R5): Anti + (R˙) → (Anti˙^+^) + (R^−^)			(R6): Anti + (R˙) → (Anti˙^−^) + (R^+^)		
	Ibu-phen	LC	Copper(ii) complex	Ibu-phen	LC	Copper(ii) complex
HOO˙	628.4 (196.8)	620.6 (146.2)	1004.3 (293.5)	1118.0 (627.4)	1267.4 (767.1)	511.5 (535.6)
CH_3_OO˙	621.2 (206.1)	613.5 (155.5)	995.0 (302.8)	995.5 (555.4)	1144.8 (695.1)	388.9 (463.6)
HO˙	563.0 (102.4)	555.2 (51.8)	936.7 (199.2)	1496.7 (972.2)	1646.0 (1111.9)	890.1 (880.4)
ABTS˙^+^	48.1 (163.3)	40.3 (112.7)	421.8 (260.0)	960.1 (441.1)	1109.4 (580.8)	353.5 (349.3)
DPPH˙	384.2 (127.1)	376.4 (76.6)	757.9 (223.9)	629.7 (294.0)	779.0 (433.7)	23.1 (202.2)

a Values in parentheses correspond to the results obtained in water solvent.

On the basis of the results in Tables 5 and 6, it can be noted that the electron-donating reactions in the gas phase for Ibu-phen and LC are thermodynamically more favorable than the electron-accepting ones with two-fold lower values of Δ*H*^0^ and Δ*G*^0^. For example, Δ*H*^0^ of the reactions (R5) and (R6) between HOO˙ and Ibu-phen increases from 632.2 to 1117.8 kJ mol^−1^, respectively, while Δ*G*^0^ value increases from 628.4 to 1118.0 kJ mol^−1^, respectively. However, for copper(ii) complex, similar Δ*H*^0^ and Δ*G*^0^ value are obtained for the same reactions. Indeed, Δ*H*^0^ and Δ*G*^0^ values of reaction (R5) are equal to 1003.4 and 1004.3 kJ mol^−1^, respectively, while the ones of reaction (R6) are two-fold lower, 511.9 and 511.5 kJ mol^−1^, in turn.

In water solvent, the reaction enthalpies and Gibbs free energies are all strongly decreased because of the high solvation enthalpy of electron. And, the same trend is also found in water solvent with the higher Δ*H*^0^ and Δ*G*^0^ values of the electron-accepting reaction (R5) than the ones of the electron-donating reaction (R6). For example, the Δ*H*^0^ value of reactions (R5) and (R6) between copper(ii) complex and ABTS˙^+^ in water solvent increases from 264.1 to 353.7 kJ mol^−1^, respectively (Table 5). These results indicate the higher electron-accepting capacity of the studied compounds by compared to their electron-donating capacity which are coherent with the IE and EA data presented in Table 4.

Furthermore, the electron-donating ability of the three studied compounds can be classified in descending order as follows: LC ≈ Ibu-phen > copper(ii) complex. For example, Δ*H*^0^ values of LC, Ibu-phen and copper(ii) complex for reaction (R5) with DPPH˙ radical in the gas phase are 385.1, 389.4 and 760.6 kJ mol^−1^, respectively, while Δ*G*^0^ values are 376.4, 384.2 and 757.9 kJ mol^−1^, in turn. The same trend is also found in water solvent with Δ*H*^0^ values of LC, Ibu-phen and copper(ii) complex are equal to 78.5, 129.2 and 225.2 kJ mol^−1^, respectively (Table 5). The corresponding Δ*G*^0^ values are 76.6, 127.1 and 223.9 kJ mol^−1^, respectively (Table 6).

Conversely, the electron-accepting capacity of the considered compounds decreases in the following trend: copper(ii) complex > Ibu-phen ≈ LC. For example, Δ*H*^0^ values of reaction (R6) with DPPH˙ radical in the gas phase are equal to 26.4, 632.3 and 777.3 kJ mol^−1^ for copper(ii) complex, Ibu-phen and LC, respectively (Table 5). Similarly, Δ*G*^0^ values for the same reaction increase from 23.1 kJ mol^−1^ for copper(ii) complex to 629.7 kJ mol^−1^ for Ibu-phen and 779.0 kJ mol^−1^ for LC (Table 6). The same results are obtained in water solvent.

This observation is consistent with the obtained results from DPPH˙, ABTS˙^+^ and HO˙ assays (Fig. 3–5), and it agrees with the conclusion obtained from UV-Vis study (Section 4.7.) in which we proposed that copper(ii) complex scavenges DPPH˙ radical by accepting one electron and then one proton particle.

Moreover, in comparing the Δ*H*^0^ and Δ*G*^0^ values of the reactions between copper(ii) complex with DPPH˙, ABTS˙^+^ and HO˙ radicals in both the gas phase and water solvent, it can be seen that the reaction feasibility trend decreases in the following order: DPPH˙ > ABTS˙^+^ > HO˙. This result is also in agreement with the experimental observations as discussed above (Table 2).

Finally, the results resumed in Tables 5 and 6 show that the electron transfer processes are strongly endogenic with highly positive values of reaction enthalpies and Gibbs free energies. While the electron-donating reactions (R5) are less endogenic than the electron-accepting ones (R6) for Ibu-phen and LC, the reaction (R6) for copper(ii) complex is more endogenic than the reaction (R5).

#### 4.9.3. Proton loss (PL) mechanism

Proton loss consists in the first step of the two-steps mechanisms including sequential proton loss electron transfer (SPLET) or proton coupled electron transfer (PCET). The difference of these mechanisms with the hydrogen transfer (HT) one is that one proton (H^+^) and one electron (e^−^) are separately donated to free radical by different channels, while a single entity (H˙) is transferred in HT mechanism.^74^ The proton loss characterized by proton affinity (PA) acts as the initiation reaction in these two-steps mechanisms. For that reason, the PA values of the ligands as well as copper(ii) complex are finally calculated in this study. In principal, the lower the PA value is, the higher the proton-donating ability of the studied compound possesses.

Table 7 resumes proton affinity (PA) values calculated for LC, Ibu-phen ligands and copper(ii) complex in the gas phase and in water solvent at the M05-2X/6-311++g(2df,2p)//M05-2X/LanL2DZ level of theory.

**Table 7:** PA value (kJ mol^−1^) for LC, Ibu-phen ligands and copper(ii) complex in the gas phase at the M05-2X/6-311++g(2df,2p)//M05-2X/LanL2DZ level of theory, (values in parentheses correspond to PA obtained in water solvent)

Proton donating position	Proton affinity (kJ mol^−1^)		
	Ibu-phen	LC	Copper(ii) complex
Ibu-C11–H	1401.0		1014.6
Ibu-C12–H	**1398.5 (249.3)**		1022.1 (226.8)
Ibu-C6–H	1457.9		
Ibu-C13–H	1401.0		1208.5
Ibu-N32–H	1400.0		
LC-C65–H		**1454.2 (461.8)**	**844.8 (336.8)**
LC-C66–H		1456.0	
LC-N57–H		1465.0	
LC-C60–H		1460.6	
LC-C56–H		1531.1	856.9

Generally, it can be seen that copper(ii) complex possesses more remarkable proton-donating ability than the two ligands Ibu-phen and LC with lower PA values in both the gas phase and water solvent. Indeed, the lowest PA values in the gas phase of Ibu-phen found at the C12–H position is equal to 1398.5 kJ mol^−1^, whereas the PA of copper(ii) complex calculated at the same position decreases to 1022.1 kJ mol^−1^. The lowest PA in the gas phase for LC ligand is equal to 1454.2 kJ mol^−1^ obtained at the C65–H position. This value is almost two times higher than the one of copper(ii) complex calculated at the same position, *i.e.* 844.8 kJ mol^−1^, which consists also in the easiest proton donating position. Similarly, PA value calculated in water solvent at the C65–H position decreases from 461.8 to 336.8 kJ mol^−1^ for LC ligand and copper(ii) complex, respectively.

Thus, it is observed that the lowest PA value of LC, Ibu-phen ligands and copper(ii) complex is classified in decreasing order: LC > Ibu-phen > copper(ii) complex with the values being 1454.2, 1384.5 and 856.9 kJ mol^−1^, respectively in the gas phase, and being 461.8, 249.3 and 226.8 kJ mol^−1^, respectively in water solvent. This result shows that the antioxidant activity *via* the proton-donating capacity of three studied compounds increases in the inverse trend: LC < Ibu-phen < copper(ii) complex.

## 5 Conclusion

A new mononuclear copper(ii) complex, [Cu(LC)(Ibu-phen)(H_2_O)_2_](ClO_4_)_2_ (LC: lidocaine, Ibu-phen: ibuprofen amide-phenanthroline), has been synthesized and characterized in order to study its antioxidant activity. The density functional theory (DFT) modeling was also investigated to characterize the structural and electronic properties of the ligands and copper(ii) complex in the gas phase and water solvent at the M05-2X/6-311++g(2df,2p)//M05-2X/LanL2DZ level of theory. ESP maps, NPA charge, HOMOs and LUMOs distributions and NBO analyses were systematically analyzed. Finally, the free radical scavenging activities in both media *via* hydrogen transfer (HT), single electron transfer (SET) and proton loss (PL) mechanisms were also computed by calculating the characterizing thermochemical properties such as BDE, IE, EA and PA quantities. The findings are multiple:

(i) Structural characterization by FT-IR spectroscopy, elemental analysis, thermogravimetric analysis and mass spectrometry shows that the ligands LC, Ibu-phen and two H_2_O molecules coordinate with Cu(ii) ion in distorted octahedral geometry in which O and N atoms of the ligands are bound to the copper ion. The structure of copper(ii) complex and the coordination environment around Cu(ii) center were also confirmed by EPR and UV-Vis spectroscopy.

(ii) Optimized structure of copper(ii) complex and ligands are calculated. ESP maps and NPA charge distributions analysis demonstrate the highly negative charges found on the heteroatoms including N53, N54 atoms of Ibu-phen and O55 and N56 atoms of LC which favor the ligands to bind with the Cu94 ion.

(iii) Frontier molecular orbitals analyses indicate that HOMO for copper(ii) complex is mainly localized at Ibu-phen ligand, while its LUMO is distributed at LC ligand. Moreover, the delocalized regions of the complex are essentially found at the phenyl moieties of two ligands. The natural bond orbital (NBO) analyses also show the electron-accepting role of the central Cu(ii) ion in coordinating with the ligands and water molecules.

(iv) The EPR spin trapping technique was also used to evaluate the antioxidant activities of LC, Ibu-phen, copper(ii) complex and ascorbic acid for various free radicals including DPPH˙, ABTS˙^+^ and HO˙. The obtained results show that the free radicals scavenging potential of the studied species is decreased as follows: copper(ii) complex > ascorbic acid > LC > Ibu-phen. All the DPPH˙, ABTS˙^+^ and HO˙ radicals scavenging assays also confirm this trend.

(v) The UV-Vis spectroscopies were investigated for the DPPH˙ scavenging assay by copper(ii) complex to evaluate thoroughly the reaction mechanism. The obtained results allow us to propose that copper(ii) complex scavenges DPPH˙ radical by a two-steps mechanism in which the radical accepts one electron and then one proton particle from the copper complex. This result is in agreement with computational calculations for single electron transfer (SET) mechanism.

(vi) Bond dissociation enthalpy (BDE) values of the compounds under study were calculated in the gas phase and water solvent to evaluate HT mechanism. It can be seen that the lowest BDE values of copper(ii) complex in water solvent are lower than the ones of Ibu-phen and LC. This result is in good agreement with the experimental results from DPPH˙ antioxidant assays.

(vii) Electron-donating and -accepting reactions of the ligands and copper(ii) complex with some representative radicals including HOO˙, CH_3_OO˙, HO˙, ABTS˙^+^ and DPPH˙ were considered in both media. It is shown that copper(ii) complex displays considerably higher radical scavenging activity than the ligands *via* its electron-accepting capacity. This activity decreases in the following trend: copper(ii) complex > Ibu-phen ≈ LC. Moreover, the reactivity of copper(ii) complex with different radicals decreases in the following order: DPPH˙ > ABTS˙^+^ > HO˙. This result agrees with the experimental observations.

(viii) Proton affinity calculation demonstrates that the proton loss ability of copper(ii) complex is considerably higher than the one of Ibu-phen and LC ligands, which is coherent with the experimental observation from DPPH˙ radical essays as well as UV-Vis spectroscopies results.

The present experimental and computational studies may hopefully contribute an effort towards the development of metallodrugs as potent antioxidants agents based on copper(ii) complex.

## Author contributions

The manuscript was written through contributions of all authors. All authors have given approval to the final version of the manuscript.

## Conflicts of interest

The authors declare no competing financial interest.

## Abbreviations

LC: Lidocaine
Ibu-phen: Ibuprofen amide-phenanthroline
ROS: Reactive oxygen species
ABTS: 2,2′-Azinobis(3-ethylbenzthiazoline-6-sulphonic acid)
DPPH: 2,2-Diphenyl-1-picrylhydrazyl
DIEA: *N*,*N*-Diisopropylethylamine
EDC-HCl: *N*-(3-Dimethylaminopropyl)-*N′*-ethylcarbodiimide hydrochloride
HOBt·*x*H_2_O: 1-Hydroxybenzotriazole hydrate
HOMO: Highest occupied molecular orbital
LUMO: Lowest unoccupied molecular orbital
HT: Hydrogen transfer
BDE: Bond dissociation enthalpy
IE: Ionization energy
EA: Electron affinity
Δ*H*: Adiabatic reaction enthalpy
Δ*G*: Gibbs free energy
NBO: Natural bond orbital
SET: Single electron transfer
PL: Proton loss
PA: Proton affinity
ZPE: Zero-point vibrational energy
EPR: Electron paramagnetic resonance
TGA: Thermogravimetric analysis
DTG: Differential thermogravimetric analysis
calcd: Calculated

## Supplementary Material

RA-009-C8RA09763A-s001
