# Supplementary material for: Experimental and theoretical evaluation on the antioxidant activity of a copper(ii) complex based on lidocaine and ibuprofen amide-phenanthroline agents
Source: RSC Adv. 2019 Jan 25;9(6):3320–35. doi: 10.1039/c8ra09763a (PMC9060296; doi:10.1039/c8ra09763a)
Supplement: RA-009-C8RA09763A-s001 [file RA-009-C8RA09763A-s001.pdf]

Electronic Supplementary Material (ESI) for RSC Advances.
This journal is © The Royal Society of Chemistry 2019

Supporting Information

Experimental and Theoretical Evaluation on the Antioxidant Activity of a Copper (II) Complex Based on Lidocaine and Ibuprofen Amide-Phenanthroline Agents

Leila Tabrizi[1*‡], Duy Quang Dao[2*‡], Thuy An Vu[3],

[1]School of Chemistry, National University of Ireland, Galway, University Road, Galway, H91 TK33, Ireland.

[2]Institute of Research and Development, Duy Tan University, 03 Quang Trung, Da Nang, 550000, Viet Nam.

[3]Faculty of Environment and Chemical Engineering, Duy Tan University, 03 Quang Trung, Da Nang, 550000, Viet Nam

Corresponding author: Leila Tabrizi; Email: LEILA.TABRIZI@nuigalway.ie

Corresponding author: Duy Quang Dao; Email: daoduyquang@gmail.com

LIST OF SUPPORTING INFORMATION

Figure S1. ^{1}H NMR spectrum of ligand ibuprofen amide-phenanthroline (Ibu-phen) (DMSO-d_6).

Figure S2. ^{13}C NMR spectrum of ligand ibuprofen amide-phenanthroline (Ibu-phen) (DMSO-d_6).

Figure S3. TOF MS spectrum of ligand ibuprofen amide-phenanthroline (Ibu-phen).

Figure S4. TOF MS spectrum of copper (II) complex.

Figure S5. FT-IR of ligand LC.

Figure S6. FT-IR of ligand Ibu-phen.

Figure S7. FT-IR of copper (II) complex

Figure S8. UV-Vis spectra of copper (II) complex in DMSO solution (**a**) and solid state (**b**).

Figure S9. UV/Vis spectra of copper (II) complex in DMSO solution at room temperature during 72h.

Table S1. Cartesian coordinates and molecular enthalpies of copper (II) complex structures for different multiplicities from 2 to 8 optimized at M05-2X/LanL2DZ level of theory.

Table S2. Natural population charges of heavy atoms in the copper (II) complex calculated at the M05-2X/6-311++g(2df,2p) level of theory.

Table S3. Natural bond orbital (NBO) analysis of the copper (II) complex calculated at the M05-2X/6-311++g(2df,2p) level of theory

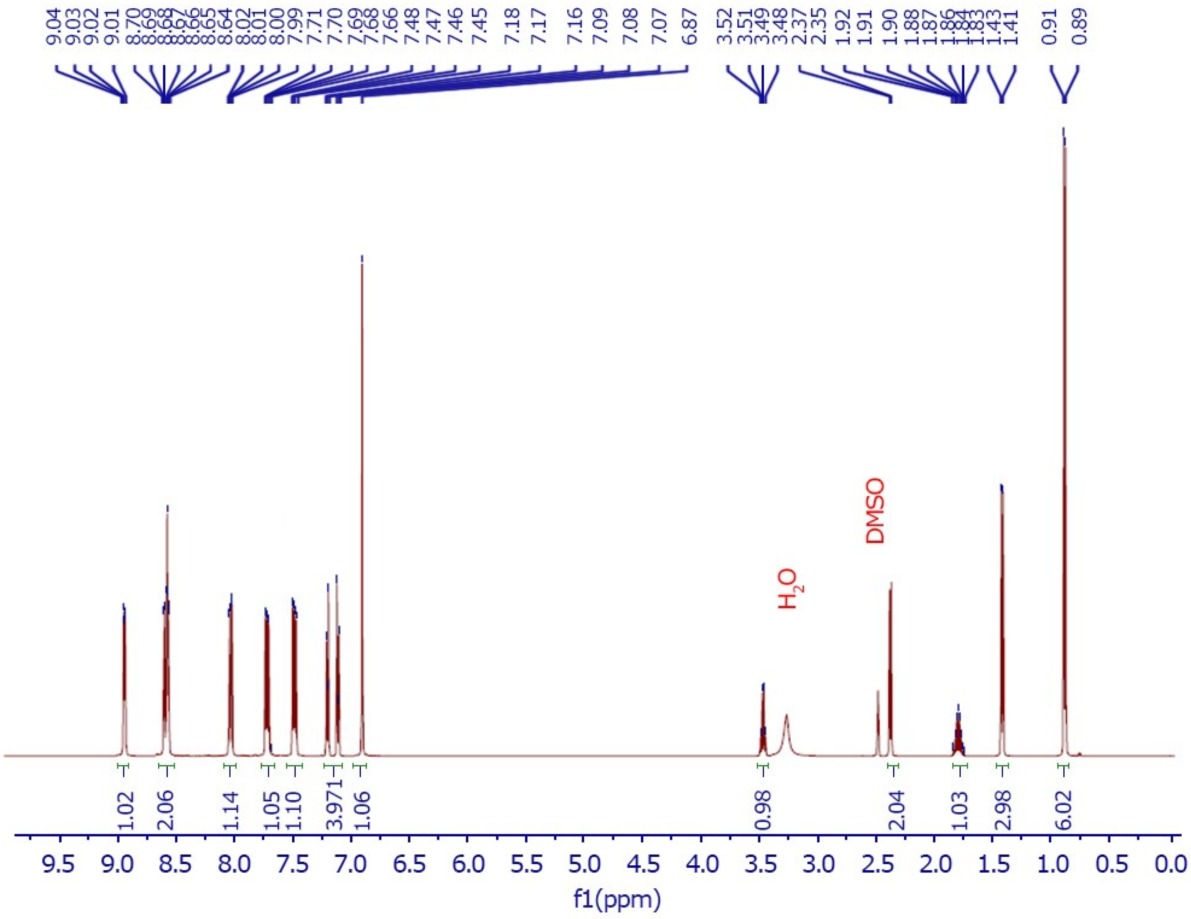

Figure S1. ^{1}H NMR spectrum of ligand ibuprofen amide-phenanthroline (Ibu-phen) (DMSO-d_6).

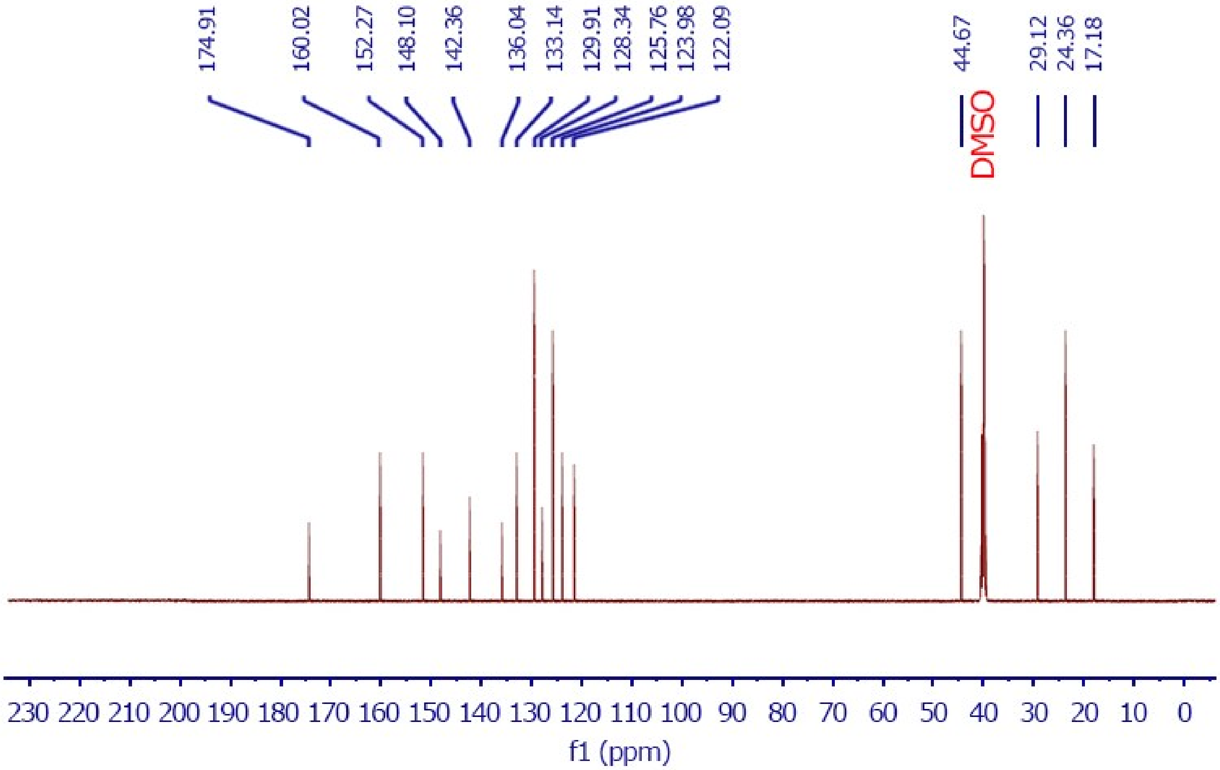

Figure S2. ^{13}C NMR spectrum of ligand ibuprofen amide-phenanthroline (Ibu-phen) (DMSO-d_6).

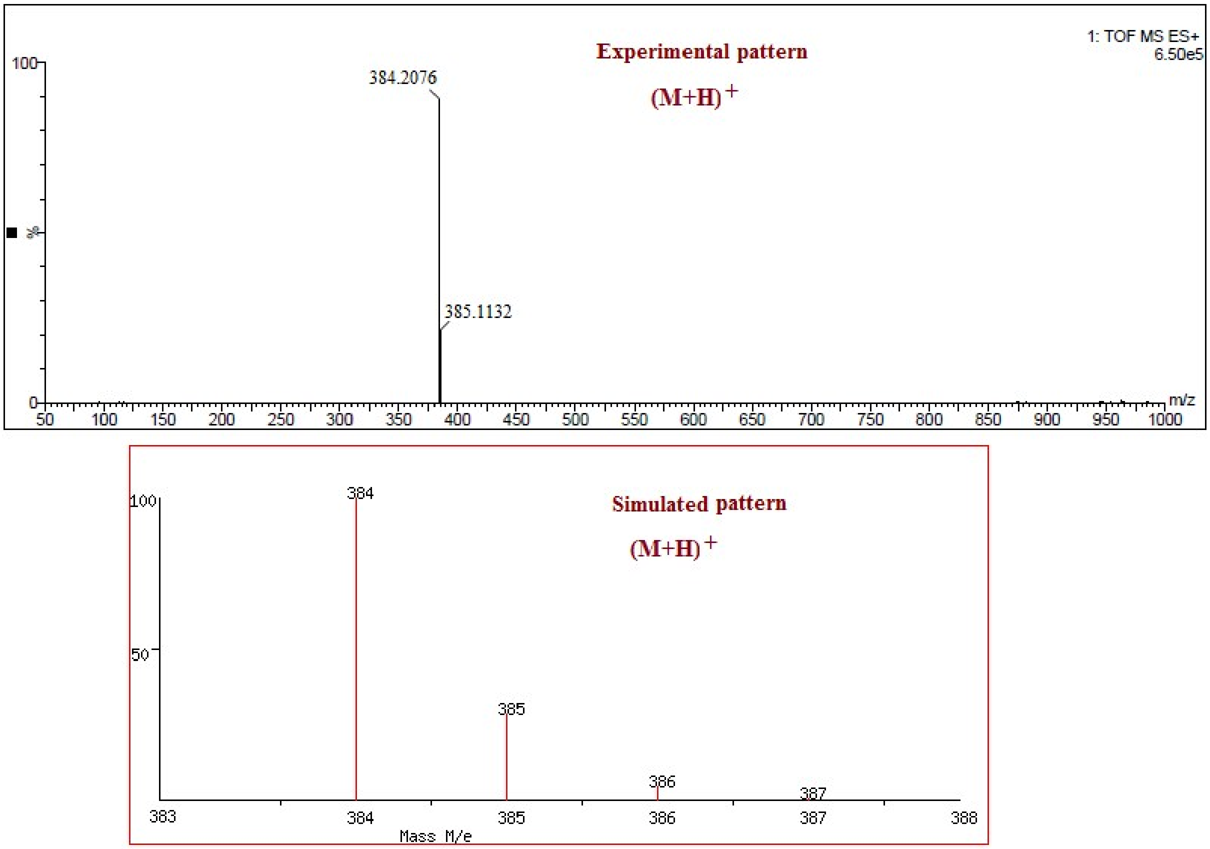

Figure S3. TOF MS spectrum of ligand ibuprofen amide-phenanthroline (Ibu-phen).

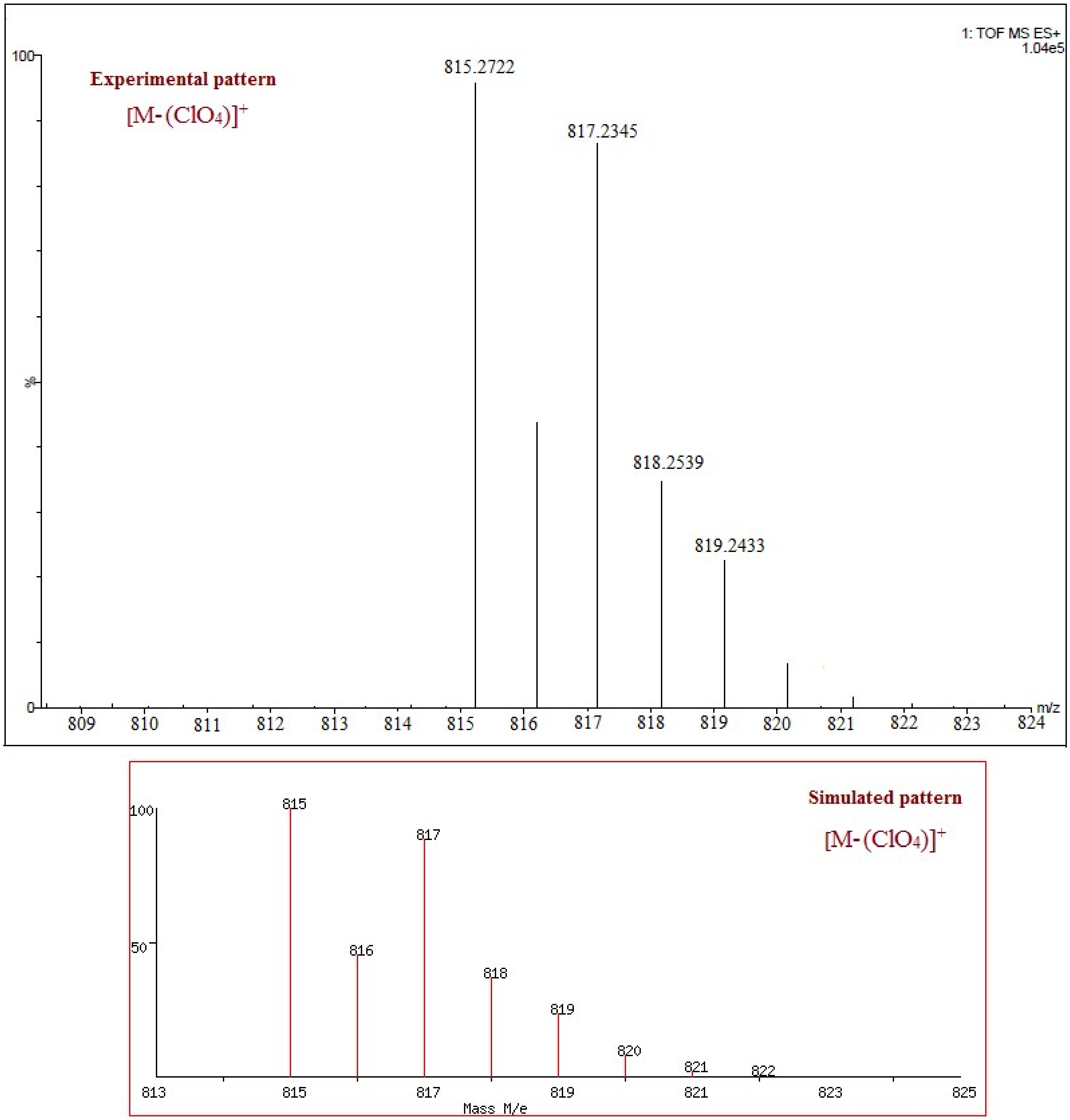

Figure S4. TOF MS spectrum of copper (II) complex.

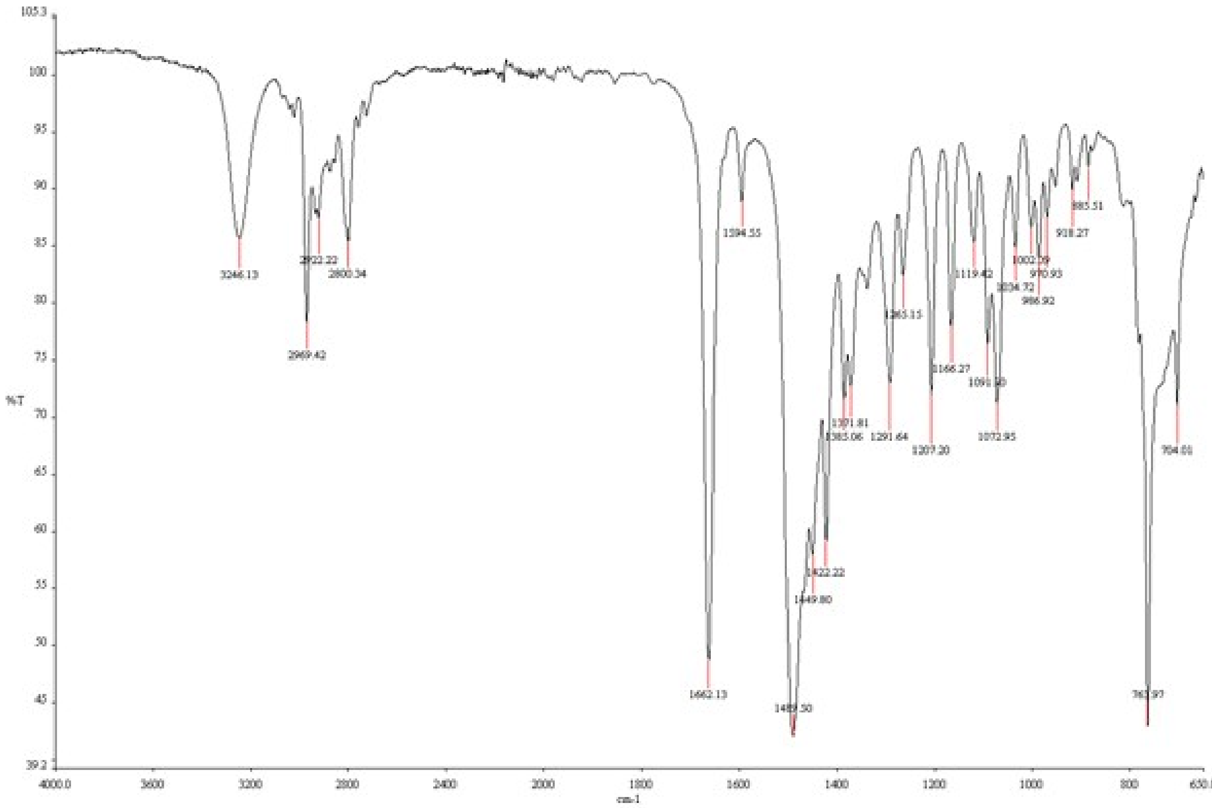

Figure S5. FT-IR of ligand LC

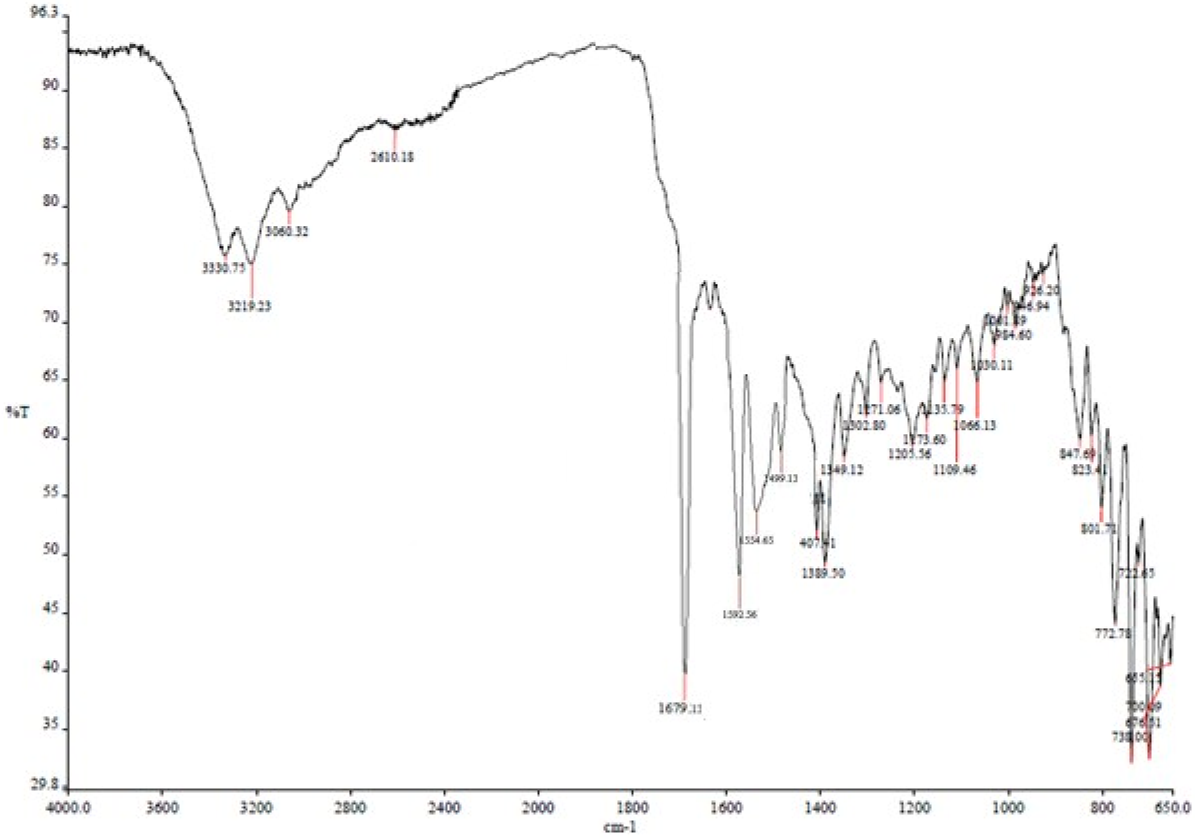

Figure S6. FT-IR of ligand Ibu-phen

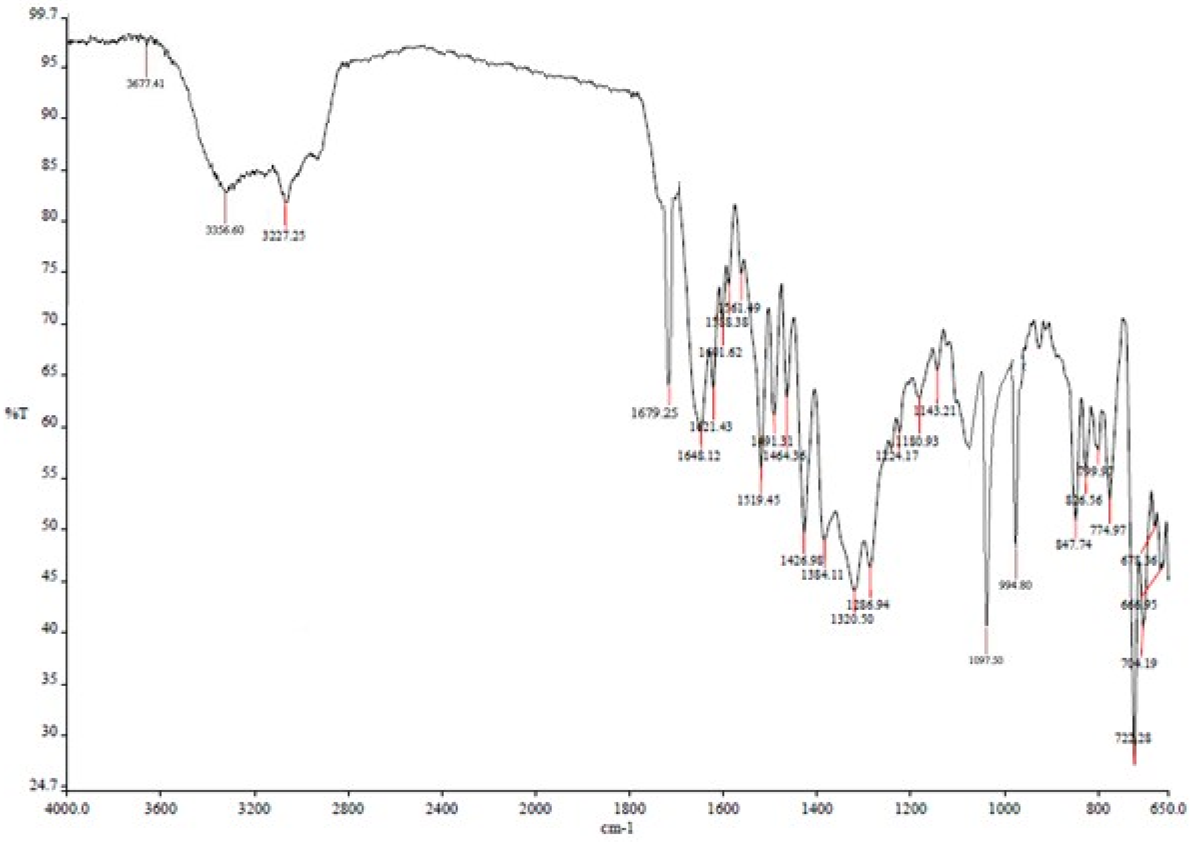

Figure S7. FT-IR of copper (II) complex

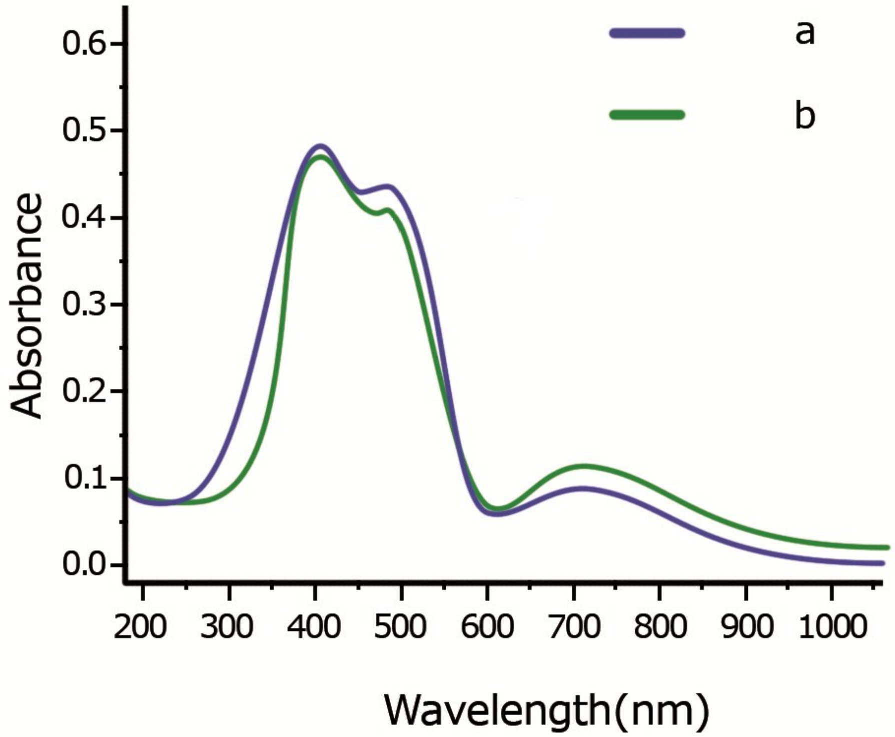

Figure S8. UV-Vis spectra of copper (II) complex in DMSO solution (**a**) and solid state (**b**).

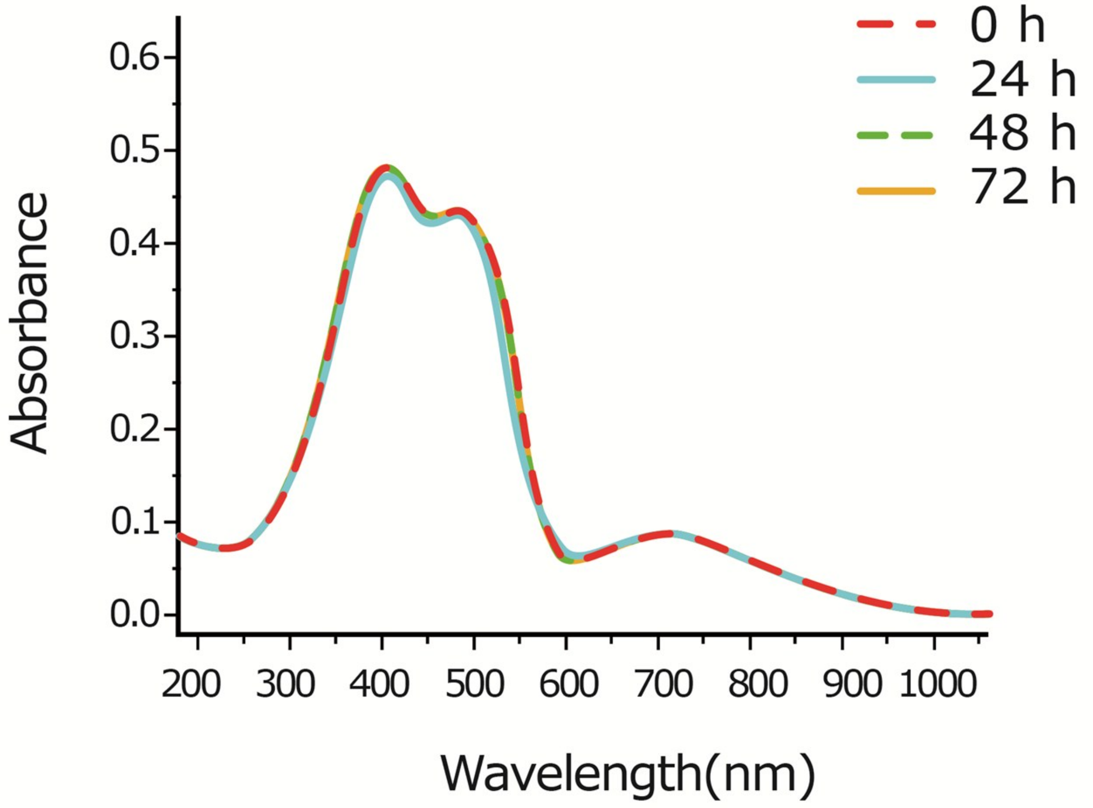

Figure S9. UV/Vis spectra of copper (II) complex in DMSO solution at room temperature during 72h.

Table S1. Cartesian coordinates and molecular enthalpies of copper (II) complex structures for different multiplicities from 2 to 8 optimized at M05-2X/LanL2DZ level of theory.

**Cartesian coordinates**				**Multiplicity = 2**
2 2				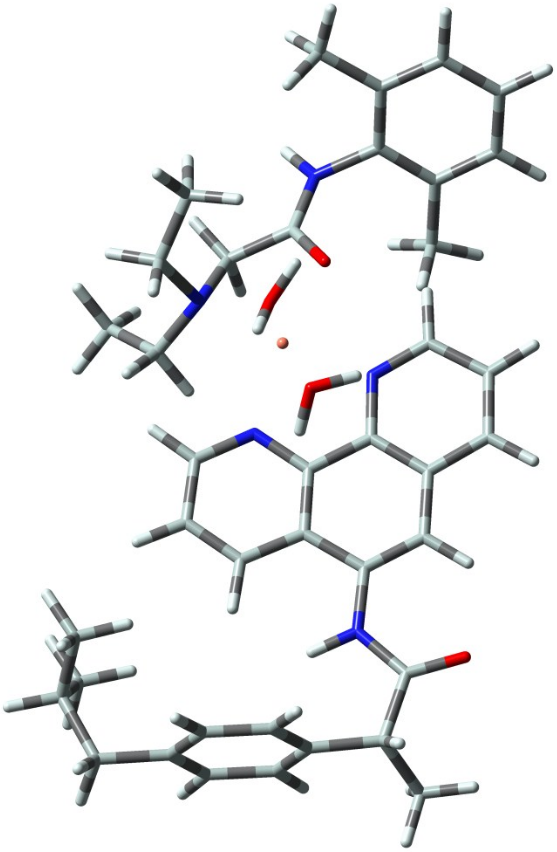
O	4.43015200	-2.94646300	-3.64407200	
C	6.64647300	3.10689200	1.57653000	
C	7.86517100	2.46505800	0.86090100	
C	7.51176400	1.33448300	-0.08049600	
C	6.87722700	-0.79984000	-1.85974900	
C	6.47210500	-1.90782800	-2.81847100	
C	5.66834400	3.74750200	0.58021700	
C	5.91997500	2.10198800	2.48337300	
C	7.46834900	0.00190700	0.36850700	
C	7.24648300	1.57906900	-1.44103500	
C	7.15412100	-1.05002400	-0.50383500	
C	6.93161800	0.53056100	-2.31483700	
C	7.15002100	-3.26846400	-2.55136000	
C	4.95723900	-2.13043200	-2.86436200	
H	7.05055600	3.90288700	2.21139200	
H	8.55725300	2.09767700	1.62508100	
H	8.38838100	3.24892200	0.30449000	
H	6.74066200	-1.60385400	-3.83549000	
H	5.22502800	2.98840200	-0.07571600	
H	6.17216700	4.48048000	-0.05620200	
H	4.86237500	4.26569600	1.11099100	
H	5.11779600	2.59728100	3.04115900	
H	5.48458900	1.28651900	1.89306400	
H	6.60435200	1.65540600	3.21028500	
H	7.70854000	-0.21624400	1.40323700	
H	7.31316300	2.59229900	-1.82131000	
H	7.15497300	-2.06613900	-0.12832000	
H	6.74910800	0.74300800	-3.36316100	
H	8.23396300	-3.14572000	-2.54535900	
H	6.84340900	-3.69397200	-1.59291200	
H	6.87589300	-3.97468100	-3.33504100	
N	4.21175500	-1.36298400	-1.97195000	
H	4.77876200	-0.75554800	-1.39564400	
C	3.07414000	0.55222200	-0.14471800	
C	2.28357200	-0.38758800	-0.85766200	
C	2.46744000	1.45233200	0.71926600	
C	0.88708400	-0.35560200	-0.62801000	
C	2.82667000	-1.34763300	-1.81625800	
C	1.07111500	1.40550800	0.89635000	
H	3.05299100	2.18679000	1.25318900	
C	0.01713000	-1.22187200	-1.37216500	
C	1.97624500	-2.17172900	-2.52399100	
H	0.58648900	2.10649400	1.55622100	

C	0.56109000	-2.10508300	-2.32435600	
H	2.38561000	-2.86465200	-3.24399200	
C	-0.35786100	-2.90136600	-3.06426700	
C	-2.18050000	-1.85783100	-1.85701300	
C	-1.71890900	-2.77169800	-2.83584800	
H	0.01658800	-3.59392000	-3.80742300	
H	-3.23299900	-1.71907100	-1.65193800	
H	-2.43736100	-3.35351900	-3.39510100	
H	4.14925200	0.60872000	-0.26584000	
N	-1.32403700	-1.11610300	-1.14670300	
N	0.29857400	0.51708600	0.25708300	
O	-3.67411300	-0.34559300	0.17104200	
N	-2.52313600	1.23193100	2.03480400	
N	-5.67673000	-0.49044500	1.27531100	
C	-3.71334700	0.42763600	2.43958700	
C	-1.52135900	1.24630100	3.16929600	
C	-2.88518800	2.66214300	1.68623900	
C	-6.38255900	-1.16681800	0.20188500	
C	-4.39209400	-0.14803600	1.21404300	
C	-7.39048000	-0.45752800	-0.47422500	
C	-6.06298900	-2.50504200	-0.08805000	
C	-2.10323500	1.52499300	4.56275200	
C	-4.17624000	2.82869500	0.88470200	
C	-8.08941400	-1.12565300	-1.49015500	
C	-6.78073300	-3.13767900	-1.11411600	
C	-7.71819300	0.97326700	-0.11445300	
C	-5.00025900	-3.25659700	0.68002600	
C	-7.78519300	-2.45429900	-1.81142600	
H	-3.35492000	-0.42668500	3.01971700	
H	-4.40583400	1.00390000	3.05963700	
H	-1.00234000	0.28679400	3.16540700	
H	-0.80108600	2.02945200	2.93537900	
H	-2.96959700	3.23461200	2.61560700	
H	-2.04772000	3.06292200	1.11313300	
H	-6.20425900	-0.25550100	2.11046600	
H	-2.66714100	2.45934200	4.60399100	
H	-2.74299000	0.71603500	4.91955100	
H	-1.27930200	1.61598500	5.27368100	
H	-4.17250600	2.23344700	-0.02786600	
H	-5.06489600	2.58132900	1.47106600	
H	-4.27084600	3.87816800	0.59841400	
H	-8.87458000	-0.60574800	-2.02454500	
H	-6.56821300	-4.17317900	-1.35084000	
H	-8.15598700	1.04952700	0.88664700	
H	-6.82804700	1.60848100	-0.14258000	
H	-8.44497000	1.38518900	-0.81423300	
H	-5.06497200	-3.06737900	1.75516400	
H	-5.10624100	-4.33026800	0.52419200	

H	-4.00064600	-2.96306300	0.33649000	
H	-8.33843000	-2.95981000	-2.59207700	
Cu	-1.75995900	0.19445300	0.33526400	
O	-1.54409600	-1.70684100	1.75248600	
H	-0.78523000	-2.01669300	2.27972800	
H	-1.98738100	-2.44890700	1.30046600	
O	-1.96814800	1.81395200	-1.22621000	
H	-2.73226600	1.78382100	-1.82972000	
H	-1.20351700	2.26070700	-1.63110900	
Energy calculation at M05-2X/LanL2DZ				**HF=-2286.8279716**

**Cartesian coordinates**				**Multiplicity = 6**
2 6				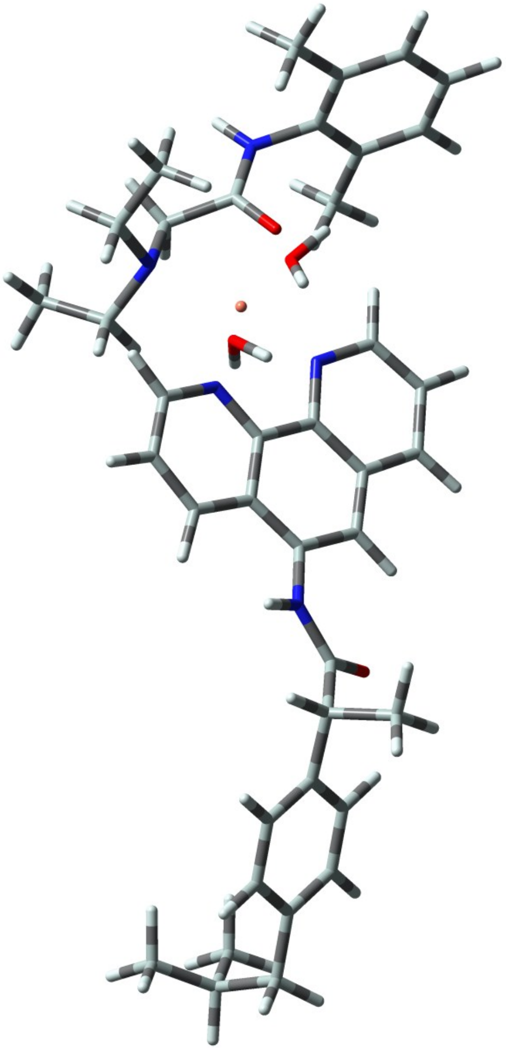
O	4.67464200	-1.71597400	-0.84696000	
C	10.85489000	0.04351200	2.32768200	
C	10.94613900	-0.45310200	0.81534700	
C	9.65028000	-0.34224400	0.11818400	
C	7.16379400	-0.12333400	-1.23399900	
C	5.91250400	-0.01679900	-2.05938700	
C	10.48403400	1.52882500	2.41899400	
C	9.90420100	-0.82852500	3.15665100	
C	8.70662800	-1.42830500	0.13221100	
C	9.29628100	0.86905900	-0.58610800	
C	7.50219300	-1.33093000	-0.52356500	
C	8.09326100	0.96981200	-1.24310600	
C	6.13261600	-0.85152700	-3.36648700	
C	4.66602800	-0.57871800	-1.36811100	
H	11.87351200	-0.09003000	2.70157000	
H	11.28216100	-1.49248800	0.84684600	
H	11.70441100	0.16515600	0.32780900	
H	5.75626100	1.02742600	-2.34056900	
H	9.44730500	1.70291200	2.11161100	
H	11.14360600	2.15300600	1.80897000	
H	10.57854700	1.86632100	3.45363600	
H	9.98142600	-0.55265500	4.21093900	
H	8.86117000	-0.68510200	2.85599200	
H	10.15106800	-1.89110600	3.07654400	
H	8.97009100	-2.33453000	0.66208800	
H	10.00443400	1.68771400	-0.59660700	
H	6.78673300	-2.14142600	-0.51369800	
H	7.84047400	1.87345300	-1.78362900	
H	7.01216700	-0.49862200	-3.90719400	
H	6.25888200	-1.90596300	-3.11724500	
H	5.25975400	-0.74694900	-4.01377900	
N	3.55409000	0.23308500	-1.42860400	
H	3.68626600	1.16856800	-1.79628600	

C	1.65380300	2.35988300	-0.92323100	
C	1.31852100	0.97977600	-0.89560500	
C	0.60367100	3.32360900	-0.86864000	
C	-0.06557600	0.62686000	-0.72491200	
C	2.24064800	-0.08217600	-1.05731500	
C	-0.70129700	2.91670600	-0.72262300	
H	0.82746500	4.37889700	-0.93677100	
C	-0.48569300	-0.73584200	-0.69603400	
C	1.80517700	-1.50435100	-1.00910700	
H	-1.51086300	3.63191400	-0.70764500	
C	0.46041900	-1.81588700	-0.85818300	
H	2.54792100	-2.28145700	-1.09524500	
C	-0.05171800	-3.15234100	-0.85887900	
C	-2.30713300	-2.28174200	-0.60065900	
C	-1.45656700	-3.35444400	-0.75170800	
H	0.62356100	-3.98895800	-0.97222400	
H	-3.38160800	-2.38229300	-0.53856200	
H	-1.86732700	-4.35335100	-0.79851500	
H	2.67949200	2.70500700	-0.94346200	
N	-1.81922700	-0.95950500	-0.52127500	
N	-1.05508300	1.57377800	-0.60053400	
O	-4.58546500	-0.20933500	-0.26343700	
N	-3.85753700	2.10359600	0.94963400	
N	-6.55206800	-0.41618800	0.88089900	
C	-4.91743400	1.27647100	1.59755400	
C	-2.83409300	2.52281200	1.98233400	
C	-4.45658700	3.30383000	0.27147000	
C	-6.99024200	-1.58229600	0.13455200	
C	-5.37303900	0.17149000	0.66814500	
C	-7.77459900	-1.39443300	-1.01424700	
C	-6.60775200	-2.85046100	0.60284500	
C	-3.38867900	3.36906100	3.13548600	
C	-5.46077400	2.94710900	-0.82298700	
C	-8.19949800	-2.53756400	-1.70794600	
C	-7.04788000	-3.97081400	-0.11746100	
C	-8.13957500	-0.00873700	-1.49215200	
C	-5.75763300	-2.99888900	1.84335200	
C	-7.84036500	-3.81607600	-1.26245200	
H	-4.46989600	0.76618400	2.45450200	
H	-5.75983800	1.89006300	1.93384400	
H	-2.39193600	1.60166400	2.36486000	
H	-2.05390600	3.07599600	1.45934500	
H	-4.94391500	3.93136000	1.02622800	
H	-3.63175800	3.88018100	-0.15245700	
H	-7.14529300	-0.07023200	1.62702100	
H	-3.72714600	4.35456600	2.81135700	
H	-4.21171500	2.87189700	3.65566900	
H	-2.59365900	3.52484800	3.86830700	

H	-4.99924600	2.33236600	-1.59615200	
H	-6.33492900	2.42594500	-0.42107500	
H	-5.82513800	3.86683100	-1.28622600	
H	-8.81431200	-2.42476900	-2.59244500	
H	-6.77747500	-4.96242300	0.22437800	
H	-8.66857200	0.55995500	-0.72187500	
H	-7.24537800	0.55863000	-1.76976100	
H	-8.78744400	-0.06378400	-2.36721200	
H	-6.28617500	-2.64208800	2.73302400	
H	-5.50699500	-4.04747400	2.00812600	
H	-4.82584600	-2.43055000	1.76395300	
H	-8.18072700	-4.68959400	-1.80359800	
Cu	-2.85279100	0.75663300	-0.37446600	
O	-2.76931600	-0.76220300	2.29347900	
H	-2.48061500	-1.27746600	3.06645400	
H	-2.30369300	-1.02485900	1.47145300	
O	-3.42497700	0.88731900	-2.49929200	
H	-4.05110700	0.16707300	-2.69915700	
H	-2.79870600	1.06639500	-3.22118400	
Energy calculation at M05-2X/LanL2DZ				HF=-2286.6231164

**Cartesian coordinates**				**Multiplicity = 8**
2 8				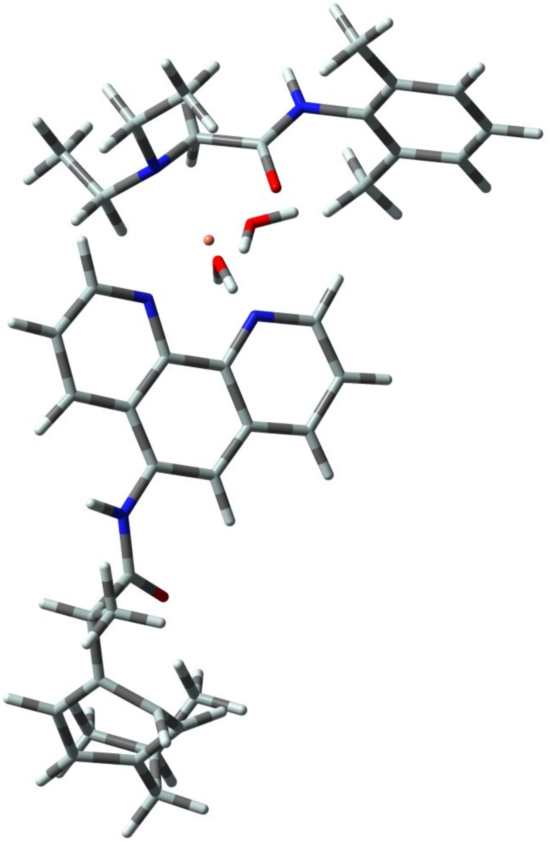
O	5.29078900	-0.40028400	-0.69638500	
C	8.60581100	-1.74648600	2.43964700	
C	9.49113200	-1.83058500	1.12291600	
C	8.92806900	-0.99806000	0.03395300	
C	7.47862500	0.63545800	-1.77232200	
C	6.19767000	1.28447400	-2.21356300	
C	8.61028100	-0.34024300	3.05085500	
C	7.17471600	-2.24926500	2.20608000	
C	7.97657300	-1.53716600	-0.90318400	
C	9.22660500	0.40364500	-0.07314500	
C	7.79671900	-0.76730200	-2.15036500	
C	8.40216100	1.21988400	-0.87075700	
C	5.99295800	1.13585100	-3.74151800	
C	5.06455700	0.59136800	-1.43321100	
H	9.11234700	-2.42931900	3.12777700	
H	9.53035100	-2.88200700	0.82796700	
H	10.49626900	-1.48933200	1.38199100	
H	6.21096300	2.34581600	-1.94063900	
H	8.04577900	0.36711000	2.43493000	
H	9.62569000	0.04228800	3.18899600	
H	8.13336800	-0.36864300	4.03358900	
H	6.65408900	-2.32135100	3.16396200	
H	6.60153000	-1.56472900	1.57166800	

H	7.16397100	-3.24617000	1.75369900	
H	7.36665700	-2.39721300	-0.65736200	
H	9.96181200	0.84084200	0.58765300	
H	8.41215200	-0.97162100	-3.02455700	
H	8.43267900	2.29714700	-0.74484000	
H	6.83222800	1.57847800	-4.28092500	
H	5.91796900	0.08123800	-4.01486600	
H	5.08310600	1.63976800	-4.07396600	
N	3.82367200	1.15474200	-1.61488900	
H	3.79377900	2.00053800	-2.17454400	
C	1.59385000	2.99896300	-1.52003300	
C	1.49563900	1.62406900	-1.17639100	
C	0.39685900	3.76502700	-1.63953000	
C	0.19071600	1.09680400	-0.87886000	
C	2.58264300	0.71782200	-1.13656300	
C	-0.82099800	3.19307700	-1.35822800	
H	0.44072900	4.80041400	-1.94667500	
C	0.00654500	-0.27441200	-0.53240900	
C	2.39326300	-0.70780900	-0.75808900	
H	-1.73947200	3.75049600	-1.47049100	
C	1.12074300	-1.19410300	-0.49016200	
H	3.25732400	-1.35093600	-0.70437100	
C	0.83949300	-2.56290900	-0.17820600	
C	-1.52950600	-2.03629400	-0.02897300	
C	-0.51146900	-2.96251800	0.02484400	
H	1.64466800	-3.28305100	-0.13430600	
H	-2.57177700	-2.29473000	0.09521100	
H	-0.74968500	-4.00020200	0.21247000	
H	2.54601300	3.49552900	-1.65548900	
N	-1.27081100	-0.66794300	-0.25917800	
N	-0.94301300	1.87176200	-0.92936300	
O	-4.12593400	-0.33918000	-0.07148700	
N	-3.79324800	2.27415500	0.56914400	
N	-6.02518000	-0.60231200	1.17114200	
C	-4.69992400	1.45380400	1.42487900	
C	-2.85396100	3.07658100	1.44316900	
C	-4.58520000	3.17668300	-0.33532100	
C	-6.26224100	-1.96300700	0.72282600	
C	-4.96377000	0.10770300	0.78393500	
C	-7.07309300	-2.17211900	-0.40369000	
C	-5.66827300	-3.01141200	1.44528700	
C	-3.54146400	4.05956300	2.39954200	
C	-5.52050800	2.42428500	-1.28017600	
C	-7.30168400	-3.49666300	-0.80593000	
C	-5.91589800	-4.32262600	1.01271300	
C	-7.67109300	-1.01024500	-1.16142300	
C	-4.80019600	-2.73330600	2.65069600	
C	-6.72889200	-4.56397500	-0.10258200	

H -4.17382500 1.23148300 2.35690300<br>H -5.63338200 1.98166700 1.64680600<br>H -2.26418000 2.34993300 2.00396900<br>H -2.17731500 3.61707300 0.78122100<br>H -5.16684500 3.87275600 0.27950700<br>H -3.86883400 3.76621700 -0.91099000<br>H -6.66586500 -0.19856400 1.84556800<br>H -4.03955300 4.87824700 1.87762900<br>H -4.26968300 3.56586800 3.04841000<br>H -2.78313400 4.50378700 3.04833400<br>H -4.96673600 1.72903500 -1.91153300<br>H -6.29778500 1.87845600 -0.73698100<br>H -6.03015200 3.14372300 -1.92503600<br>H -7.93174600 -3.69005200 -1.66536000<br>H -5.48054700 -5.15273000 1.55557600<br>H -8.28921800 -0.37981200 -0.51564300<br>H -6.88811500 -0.37830800 -1.59281000<br>H -8.30082600 -1.36810000 -1.97618200<br>H -5.37885600 -2.27860900 3.46102900<br>H -4.37316300 -3.66152200 3.03232100<br>H -3.97996400 -2.05051300 2.40870100<br>H -6.91957100 -5.58118900 -0.41983400<br>Cu -2.57920600 0.84463000 -0.46060400<br>O -2.24405000 -0.00060600 2.46841600<br>H -1.87476800 -0.27862900 3.32430900<br>H -1.73835200 -0.35465600 1.70628100<br>O -3.16158700 0.40643300 -2.53840000<br>H -3.66338900 -0.42904400 -2.56360500<br>H -2.58292100 0.53382000 -3.30928200	
Energy calculation at M05-2X/LanL2DZ	HF=-2286.4879208

Table S2. Natural population charges of heavy atoms in the copper (II) complex calculated at the M05-2X/6-311++g(2df,2p) level of theory.

Atom	No	Natural charge	Spin Density	Atom	No	Natural charge	Spin Density
O	1	-0.6326	0.0001	C	46	-0.1016	-0.0044
C	2	-0.2374	0.0000	C	47	0.0846	-0.0056
C	3	-0.4144	0.0000	C	48	-0.2267	0.0065
C	4	-0.0314	0.0000	N	53	-0.5900	0.0626
C	5	-0.0906	-0.0001	N	54	-0.6018	0.0479
C	6	-0.3318	0.0000	O	55	-0.7547	0.0500
C	7	-0.5910	0.0000	N	56	-0.6248	0.0516
C	8	-0.5898	0.0000	N	57	-0.5870	-0.0018
C	9	-0.2025	0.0000	C	58	-0.2762	0.0049
C	10	-0.2014	0.0000	C	59	-0.1695	0.0001
C	11	-0.2202	0.0000	C	60	-0.1749	0.0031
C	12	-0.2061	0.0000	C	61	0.1003	0.0021
C	13	-0.5767	0.0000	C	62	0.7631	0.0011
C	14	0.7535	0.0000	C	63	-0.0210	-0.0006
N	32	-0.6613	0.0002	C	64	-0.0293	-0.0004
C	34	-0.0842	-0.0033	C	65	-0.6212	0.0012
C	35	-0.1364	0.0026	C	66	-0.6187	-0.0005
C	36	-0.1993	0.0039	C	67	-0.1958	0.0004
C	37	0.3058	-0.0040	C	68	-0.2054	-0.0002
C	38	0.1991	0.0003	C	69	-0.6123	0.0001
C	39	0.0924	-0.0005	C	70	-0.6071	0.0000
C	41	0.2692	-0.0001	C	71	-0.1614	0.0000

C	42	-0.1792	0.0003	Cu	94	1.1682	0.7791
C	44	-0.1186	0.0030	O	95	-0.9495	0.0003
				O	98	-0.9486	0.0000

Table S3. Natural bond orbital (NBO) analysis of the copper (II) complex calculated at the M05-2X/6-311++g(2df,2p) level of theory

Donor NBO (i)	Acceptor NBO (j)	E(2), kJ/mol
LP(1) N53	LP*(5) Cu94	120.20
LP(1) N53	LP*(6) Cu94	49.60
LP(1) N53	LP*(8) Cu94	52.70
LP(1) N54	LP*(5) Cu94	40.30
LP(1) N54	LP*(6) Cu94	98.90
LP(1) N54	LP*(7) Cu94	37.10
LP(1) O55	LP*(6) Cu94	94.2
LP(1) O55	LP*(7) Cu94	50.00
LP(1) O55	LP*(8) Cu94	32.30
LP(1) O55	LP*(9) Cu94	395.8
LP(2) O55	LP*(6) Cu94	166.00
LP(2) O55	LP*(7) Cu94	82.40
LP(2) O55	LP*(8) Cu94	26.20
LP(2) O55	LP*(9) Cu94	223.70
LP(3) O55	LP*(9) Cu94	47.00
σ(1) N56–C59	LP*(9) Cu94	237.1
LP(1) N56	LP*(5) Cu94	73.8
LP(1) N56	LP*(6) Cu94	57.6
LP(1) N56	LP*(8) Cu94	35.9
σ(1) O95–H96	LP*(9) Cu94	28.7
σ(1) O95–H97	LP*(9) Cu94	26.9
LP(2) O95	LP*(9) Cu94	81.3
σ(1) O98–H99	LP*(6) Cu94	37.1
LP(2) O98	LP*(6) Cu94	124.1
LP(2) O98	LP*(9) Cu94	844.9
